# A systematic review and meta-analysis of short-stay programmes for total hip and knee replacement, focusing on safety and optimal patient selection

**DOI:** 10.1186/s12916-023-03219-5

**Published:** 2023-12-21

**Authors:** Danielle Berkovic, Patrick Vallance, Ian A. Harris, Justine M. Naylor, Peter L. Lewis, Richard de Steiger, Rachelle Buchbinder, Zanfina Ademi, Sze-Ee Soh, Ilana N. Ackerman

**Affiliations:** 1 School of Public Health and Preventive Medicine, Monash University, 553 St Kilda Road, Melbourne, VIC 3004 Australia; 2 Department of Physiotherapy, School of Primary and Allied Health Care, Monash University, Melbourne, Australia; 3 School of Clinical Medicine, UNSW Medicine and Health, UNSW Sydney, Kensington, Australia; 4 Whitlam Orthopaedic Research Centre, Ingham Institute for Applied Medical Research, Liverpool, NSW Australia; 5 Liverpool Hospital, Liverpool, NSW Australia; 6 Australian Orthopaedic Association National Joint Replacement Registry, Adelaide, Australia and Faculty of Medicine, University of Adelaide, Adelaide, Australia; 7 Department of Surgery, Epworth HealthCare, University of Melbourne, Melbourne, Australia; 8 Health Economics and Policy Evaluation Research (HEPER), Faculty of Pharmacy and Pharmaceutical Sciences, Monash University, Melbourne, Australia

**Keywords:** Enhanced recovery after surgery, Fast-track, Hip arthroplasty, Hip replacement, Knee arthroplasty, Knee replacement, Models of care, Safety, Short-stay joint replacement, Systematic review

## Abstract

**Background:**

Short-stay joint replacement programmes are used in many countries but there has been little scrutiny of safety outcomes in the literature. We aimed to systematically review evidence on the safety of short-stay programmes versus usual care for total hip (THR) and knee replacement (KR), and optimal patient selection.

**Methods:**

A systematic review and meta-analysis. Randomised controlled trials (RCTs) and quasi-experimental studies including a comparator group reporting on 14 safety outcomes (hospital readmissions, reoperations, blood loss, emergency department visits, infection, mortality, neurovascular injury, other complications, periprosthetic fractures, postoperative falls, venous thromboembolism, wound complications, dislocation, stiffness) within 90 days postoperatively in adults ≥ 18 years undergoing primary THR or KR were included. Secondary outcomes were associations between patient demographics or clinical characteristics and patient outcomes. Four databases were searched between January 2000 and May 2023. Risk of bias and certainty of the evidence were assessed.

**Results:**

Forty-nine studies were included. Based upon low certainty RCT evidence, short-stay programmes may not reduce readmission (OR 0.95, 95% CI 0.12–7.43); blood transfusion requirements (OR 1.75, 95% CI 0.27–11.36); neurovascular injury (OR 0.31, 95% CI 0.01–7.92); other complications (OR 0.63, 95% CI 0.26–1.53); or stiffness (OR 1.04, 95% CI 0.53–2.05). For registry studies, there was no difference in readmission, infection, neurovascular injury, other complications, venous thromboembolism, or wound complications but there were reductions in mortality and dislocations. For interrupted time series studies, there was no difference in readmissions, reoperations, blood loss volume, emergency department visits, infection, mortality, or neurovascular injury; reduced odds of blood transfusion and other complications, but increased odds of periprosthetic fracture. For other observational studies, there was an increased risk of readmission, no difference in blood loss volume, infection, other complications, or wound complications, reduced odds of requiring blood transfusion, reduced mortality, and reduced venous thromboembolism. One study examined an outcome relevant to optimal patient selection; it reported comparable blood loss for short-stay male and female participants (*p* = 0.814).

**Conclusions:**

There is low certainty evidence that short-stay programmes for THR and KR may have non-inferior 90-day safety outcomes. There is little evidence on factors informing optimal patient selection; this remains an important knowledge gap.

**Supplementary Information:**

The online version contains supplementary material available at 10.1186/s12916-023-03219-5.

## Background

The demand for total hip (THR) and knee replacement (KR) surgeries is increasing with the growing burden of osteoarthritis [1–3]. Between 2009 and 2019, the average rate of THR and KR increased by 22 and 35%, respectively, across all Organisation for Economic Co-operation and Development (OECD) countries [4]. The Australian Orthopaedic Association National Joint Replacement Registry (AOANJRR) [5] and the United Kingdom (UK) National Joint Registry [6] have each reported that delays in accessing joint replacement surgery, combined with anticipated growing demand, need to be addressed.

The capacity to meet expected joint replacement demand requires safe and efficient models of care. Short-stay programmes (also known as ‘fast track’, ‘enhanced recovery after surgery’ or ‘rapid recovery’ programmes) are those which seek to reduce acute hospital length of stay after elective primary THR or KR surgery [7]. Short-stay programmes utilise a clinical pathway that enhances functional recovery and facilitates earlier patient discharge. Features of these programmes may include (but are not limited to) pre-operative education, standardised anaesthetic protocol and/or utilisation of local anaesthesia, postoperative nausea prophylaxis, blood conservation measures and multimodal analgesia [8–10].

Short-stay programmes have been successfully implemented in the United States (US) and some European countries [11, 12] yet they remain underutilised in Australia [9, 13]. Systematic reviews have found that short-stay programmes for THR and KR are associated with reduced healthcare and patient costs [14], yet few controlled trials have been conducted on their safety. Reviews of short-stay programmes have thus far focused on a limited set of safety outcomes compared to usual care: one review reported fewer complications with short-stay programmes [15], and another found no effect on complications or hospital readmission [8]. Factors associated with poorer patient outcomes have not been systematically examined, yet this information is essential for guiding appropriate patient selection. Establishing the safety profile of short-stay programmes, and factors associated with suboptimal outcomes could inform future efforts to develop, implement and scale-up short-stay joint replacement programmes in Australia and other countries where these are not commonly used.

This study aimed to systematically review the evidence on the safety of short-stay THR and KR programmes, compared with usual care, and patient factors associated with poor outcomes.

## Methods

### Design

This study is a systematic review. The protocol was registered on the PROSPERO International Prospective Register of Systematic Reviews (registration number 351026) and is reported according to the Preferred Reporting Items for Systematic Reviews and Meta-Analysis (PRISMA) statement (Supplementary File 1) [16]. The second component of the registered review protocol (comprising a review of qualitative studies examining barriers and enablers to the implementation and sustainability of short-stay joint replacement programmes) will be reported separately.

### Eligibility criteria

We included randomised controlled trials (RCTs) as well as registry, interrupted time series, and other observational studies. The studies could have been conducted in any setting that compared a short-stay programme for adults aged ≥ 18 years undergoing unilateral or bilateral, total or uni-compartmental KR or THR, included a usual care (traditional or standard care) comparator group, and reported safety outcomes within 90 days post-operatively, and/or associations between patient demographics and/or clinical characteristics and patient outcomes. Short-stay programmes were those that specifically identified as being ‘short-stay’, ‘enhanced recovery after surgery’, ‘enhanced recovery’, ‘fast-track’, ‘accelerated discharge’, ‘early discharge’ or ‘rapid recovery’ programmes or models of care. A standardised definition of a short-stay programme was not adopted as such programmes are not delivered consistently across hospital settings or countries, and length of stay targets are variable. There was no study size restriction, but we excluded studies not published in English.

Exclusions were studies that only compared the partial implementation of a short-stay programme (representing the use of short-stay programme components rather than a usual care comparator) with full implementation of the programme, reviews, conference publications, case studies and grey literature. Studies reporting solely on joint replacement for traumatic injury (including traumatic fracture) or malignancy, or studies reporting solely on revision joint replacement were also excluded. Where studies involved mixed cohorts of patients, these were only eligible for inclusion if data for patients undergoing primary elective joint replacement were reported separately. Studies that focused on same-day discharge or outpatient joint replacement programmes were excluded as these patient populations are highly selected (these programmes are appropriate for a relatively small proportion of patients, based on clinical, social and home environment factors) and these types of programmes do not feature prominently in the Australian healthcare system.

### Outcomes

Safety outcomes and patient-related outcomes were selected based on discussions with the multidisciplinary research team, which comprised expertise in orthopaedic surgery, rheumatology, public health, physiotherapy, health economics and consumer-led research.

Fourteen safety outcomes were included: (1) readmissions, (2) reoperations, (3) blood loss (including requiring a blood transfusion), (4) emergency department visits, (5) infection, (6) mortality, (7) neurovascular injury, (8) other complications (when not specifically defined), (9) periprosthetic fractures, (10) postoperative falls, (11) venous thromboembolism (deep vein thrombosis (DVT) or pulmonary embolism (PE)), (12) wound complications, (13) dislocation and (14) stiffness and/or manipulation. Blood loss was measured in millilitres (ml) and the remaining outcomes had dichotomous responses (yes/no).

Six patient-related outcomes were considered in relation to patient demographics or clinical characteristics: (1) activities of daily living, including ambulation and mobility, (2) functional outcomes, (3) joint-specific patient-reported outcome measures (PROMs), (4) pain at rest or during activity, (5) patient satisfaction and (6) quality of life.

### Search strategy and identification and selection of included papers

An electronic literature search was undertaken in Medline (OVID), Cumulative Index to Nursing and Allied Health Literature (CINAHL), EMBASE and the Cochrane Central Register of Controlled Trials (CENTRAL). A comprehensive search strategy was designed using relevant search terms (Supplementary File 2). The reference lists of the included studies were hand searched to identify any additional primary studies. The search strategy was limited to articles published between 2000 and August 2022. We ran an updated search from 2022 to May 2023 before finalising the review for publication. We also searched ClinicalTrials.gov to identify current research and any published results on short-stay THR or KR programmes [17].

The retrieved articles were loaded into Covidence software (Veritas Health Innovation Ltd, Melbourne, Australia). Two review authors (IA, PV) independently screened the titles and abstracts of all retrieved studies to determine eligibility. The full texts of all potentially eligible studies were reviewed independently by the same two review authors to determine final inclusion. At each review stage, discordance regarding eligibility was discussed and resolved through consensus. Where agreement could not be reached, a third reviewer (RB) was consulted.

### Assessment of risk of bias

The risk of bias was assessed independently by two reviewers (IA, DB) using validated critical appraisal tools from the Joanna Briggs Institute (JBI). The JBI critical appraisal tools included nine items for quasi-experimental studies, and 13 items for RCTs [18]. The following domains were assessed for RCTs and each potential source of bias was judged as low risk or high risk based on yes/no/unclear (yes low risk, no and unclear high risk) responses to the items: selection and allocation, administration of intervention/exposure, assessment, detection and measurement of the outcome, participant retention and statistical conclusion validity [19]. The following domains were assessed for the quasi-experimental studies and each potential source of bias was judged using the same methods: the temporal relationship of the variables (whether it is clear that the intervention precedes the outcome), selection bias, control group, multiple measurements of the outcome, loss to follow-up and statistical conclusion validity [20].

### Data extraction and management

One review author (DB) independently extracted data from the included studies using a customised spreadsheet. A second author (IA) independently extracted a random 10% sample of these data to check for consistency. Data extracted included the study design, country, surgical procedure, gender, age, intervention components (mapped to the Consensus statement for perioperative care in THR and KR: Enhanced Recovery After Surgery (ERAS®) Society recommendations) [21], and relevant outcomes concerning the safety profile and associations between patient factors and surgical outcomes. For studies that contained more than one short-stay group (for example, partial implementation of short-stay components, full implementation of short-stay components and a usual care group), only the data for the full implementation group was extracted and compared with the usual care group.

### Data analysis

Study characteristics and demographic data were reported using means (standard deviation (SD)), medians (interquartile range (IQR)) or frequencies as appropriate. Any data on associations between patient factors and outcomes were reported as published, without further analysis. The diversity of included studies was assessed in terms of study design, interventions and outcomes to determine whether a meta-analysis was appropriate. When pooling was appropriate, data were combined according to study design to examine outcomes based on the level of evidence. Between-study variability was assessed using the *I*^2^ statistic. The *I*^2^ values were interpreted based on the Cochrane Handbook for Systematic Reviews of Interventions (0–40% may be important, 30–60% moderate, 50–90% substantial, > 75% considerable) [22]. Where both adjusted and unadjusted effect estimates were reported, we used the unadjusted estimate for meta-analysis.

Where meta-analysis was not possible due to significant diversity of outcome measures or only one study reporting a particular outcome, relevant outcome data were extracted and reported as published. Safety outcomes for THR and KR were combined for meta-analysis except for dislocation (relevant only for THR) and stiffness and/or manipulation (relevant only for KR).

For the number of events or binary outcomes, a random effects model was used and odds ratios (ORs) with 95% confidence intervals (CIs) were calculated. The Mantel–Haenszel method was used to weight each estimate. For continuous outcomes, a random effects model was used and the mean difference with 95% confidence intervals was calculated. The generic inverse variance method was used to weight each estimate. A random effects model was chosen to allow for observed differences in study results that may be due to a combination of chance and some genuine variation in the intervention effects [22]. All analyses were conducted using Review Manager, V.5.4 (Revman, The Cochrane Collaboration; Oxford, UK).

### Grading the certainty of evidence

The certainty of the evidence was assessed separately for the RCTs, followed by the registry, interrupted time series and other observational studies by two reviewers (DB, IA). The certainty of the RCT evidence was assessed using the five GRADE considerations (risk of bias, consistency of effect, indirectness, imprecision and publication bias). The methods described in the Cochrane Handbook for Systematic Reviews of Interventions were followed [23]. In accordance with the GRADE handbook, quasi-experimental studies (registry, interrupted time series and other observational studies) were initially graded as low certainty evidence and downgraded for imprecision, indirectness, inconsistency or publication bias, or upgraded if a large magnitude of effect or dose–response gradient was found [24]. The summary of findings table is presented for the RCTs only.

## Results

**Fig. 1:**
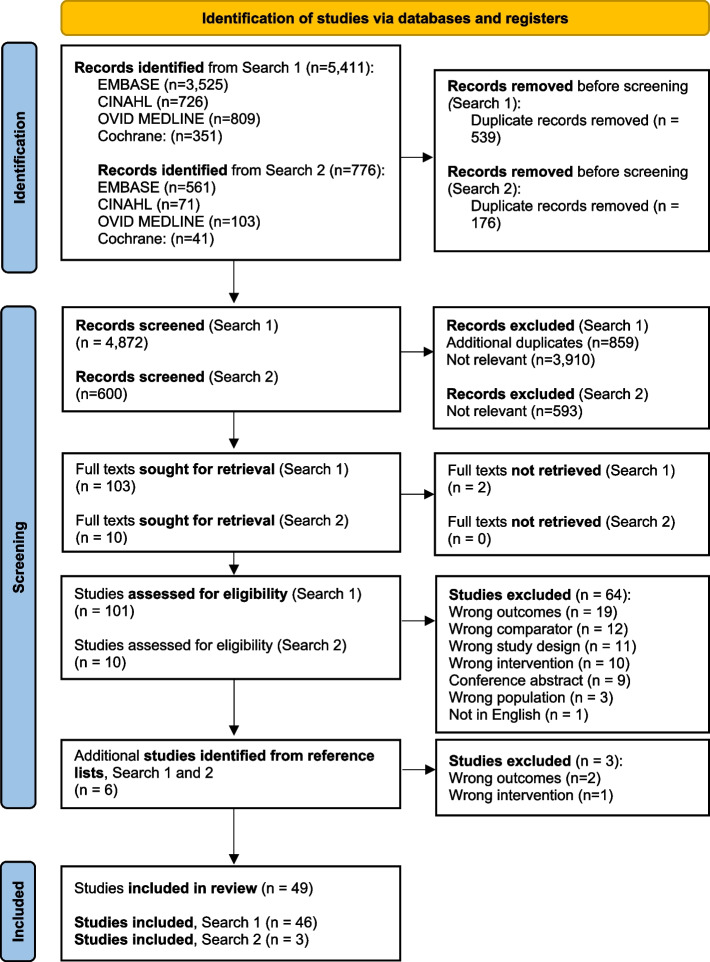
PRISMA 2020 flow diagram

Figure 1 summarises the search and screening processes. The electronic search (2000–August 2022) identified 5411 studies for potential inclusion. After duplicates were removed, 4872 titles and abstracts were screened, 101 full-text articles were screened and overall 46 studies were included. The search was updated (2022–May 2023). An additional 776 studies were identified for potential inclusion; after duplicates were removed, 602 titles and abstracts were screened, seven full-text articles were screened and overall three studies were included. We also identified four trials published on ClinicalTrials.gov; none have published results thus far.

### Trial design, setting, and characteristics

The study and participant characteristics of the 49 included studies as well as a description of the short-stay and usual care groups are shown in Table 1. They were published from 2005 to 2023 and originated from sixteen countries: thirteen from the United Kingdom (UK) [25–37], seven from the United States (US) [38–44], four from China [45–48], three studies each from France [49–51], Italy [52–54] and the Netherlands [55–57], two studies each from Canada [58, 59], Denmark [60, 61], New Zealand [62, 63], and Sweden [64, 65], and one study each from Australia [66], Brazil [67], Germany [68], India [69], Ireland [70], Norway [71], Spain [72] and Switzerland [73].

**Table 1:** Study and participant characteristics

First author, year of publication	Country	Surgical procedure (% intervention group)	Number of participants: n	% Female	Mean (SD) or median [IQR] age in years	Brief description of short-stay intervention	Brief description of usual care	Maximum study follow-up time
**Randomised controlled trials (parallel arm design)**								
Fransen et al. 2018 [57]	Netherlands	TKR	Short-stay: 25 Usual care: 24	Short-stay: 56 Usual care: 37	Short-stay: 64 (9) Usual care: 61 (7)	- Mean LoS 2 days - No tourniquet, intra-operative local infiltration and analgesia, no pain pumps, wound drains, or bladder catheters were used	- Mean LoS 4 days - Regular hospital TKR protocol, including the use of a tourniquet, wound drains, and bladder catheter	12 weeks
Petersen et al. 2006 [61]	Denmark	THR	Short-stay: 27 Usual care: 30	Short-stay: 56 Usual care: 47	Median [range] Short-stay: 55 [28–84] Usual care: 58 [26–81]	- No difference found in the LoS - Received an optimisation pack involving pre- and post-operative strategies in addition to usual care	- No difference found in the LoS - Standard pre- and post-operative multimodal anaesthesia and analgesia	30 days
Reilly et al. 2005 [33]	United Kingdom	Unicompartmental knee replacement	Short-stay: 21 Usual care: 20	Short-stay and usual care: 41	Short-stay and usual care: 63	- Mean Los 1.5 days - Facilitated discharge and discharge support in addition to usual care	- Mean LoS 1.5 days - Standard surgical, anaesthetic, and analgesic protocol	6 weeks
**Registry studies**								
Berg et al. 2018 [65]	Sweden	THR (56) TKR (44)	Short-stay: 7,270 Usual care: 6,640	Short-stay: 57 Usual care: 57	Short-stay: 70 (10) Usual care: 70 (10)	- Median LoS 3 days for THR and TKR - Admission on the day of surgery, early mobilisation, functional discharge, LoS ≤ 3 days	- Median LoS 5 days for THR and TKR - Minimum of written and oral patient information, multimodal analgesia, tranexamic acid	3 months
Berg et al. 2021 [64]	Sweden	THR (53) TKR (47)	Short-stay: 67,672 Usual care: 48,621	Short-stay: 56 Usual care: 58	Short-stay THR: 68 (10) Usual care THR: 69 (10) Short-stay TKR: 69 (9) Usual care TKR: 70 (9)	- Median LoS 3 days for THR and TKR - Admission on the day of surgery, early mobilisation, functional discharge, LoS ≤ 3 days	- Median LoS 5 days for THR and TKR - Minimum of written and oral patient information, multimodal analgesia, tranexamic acid	90 days
**Interrupted time series studies**								
Alvis et al. 2021 [38]	United States	THR (33) TKR (67)	Short-stay: 186 Usual care: 96	Short-stay: 9 Usual care: 12	Short-stay: 65 (9) Usual care: 63 (9)	- Median LoS 2 days - Involvement of the Anaesthesia Perioperative Care Service for patients in the presurgical and post-discharge period	- Median LoS 3 days - Preoperative, intraoperative, and postoperative patient care in the hospital only	30 days
Amlie et al. 2016 [71]	Norway	THR	Short-stay: 239 Usual care: 4,167	Short-stay: 72 Usual care: 67	Female: 71 (10) Male: 67 (11)	- LoS not reported - Four main components: (1) local anaesthetic, (2) cessation of negative vacuum suction drain, (3) early mobilisation, (4) standardised pain management	- LoS not reported - Patients who received only the standardised pain management protocol	3 days
Arshad et al. 2014 [25]	England	THR (50) TKR (50)	Short-stay: 48 Usual care: 48	Short-stay: 40 Usual care: 65	Short-stay: 76 [68–82] Usual care: 75 [67–82]	- Median LoS 4 days - Preoperative education, regional anaesthesia, early mobilisation, and avoidance of drains and catheters	- Median LoS 5 days - No education on patient LoS, post-operative physiotherapy commenced the day after surgery	6 weeks
Azam et al. 2022 [69]	India	Bilateral TKR	Short-stay: 275 Usual care: 190	Short-stay: 72 Usual care: 66	Short-stay: 66 (9) Usual care: 66 (10)	- Median LoS 3.9 days - 9 evidence-based interventions across the pre-operative, intraoperative, and postoperative period	- Median LoS 7.5 days - No usual care patients had any peripheral nerve blocks post-operatively, and epidural catheters were left in place for 24 h	6 weeks
Chung et al. 2021 [45]	China	THR (47) TKR (53)	Short-stay: 111 Usual care: 117	Short-stay: 60 Usual care: 67	Short-stay: 70 (9) Usual care: 68 (12)	- Mean LoS 3.28 days - Higher dose of IV steroids, optimised management of pain, nausea and vomiting, blood management, sleep management, and same-day rehabilitation	- Mean LoS 5.16 days - Usual care patients received generally similar treatment to short-stay patients (except for the specific short-stay components listed), but these treatments are not described	30 days
de Carvalho Almeida et al. 2021 [67]	Brazil	THR including same-day bilateral surgery	Short-stay: 47 Usual care: 51	Short-Stay: 57 Usual care: 51	*n* (%) Short-stay: ≤ 49: 15 (32) Usual care: ≤ 49: 13 (25) Short-stay: 50–59: 16 (34) Usual care: 50–59: 17 (33) Short-stay: ≥ 60: 16 (34) Usual care: ≥ 60: 21 (41)	- Mean LoS 2.3 days - Patient education and multidisciplinary care mandatory, the introduction of tranexamic acid, no opioids or bladder catheters, peri-articular injection, no ICU support, and early mobilisation	- Mean LoS 6.4 days - No detailed information about the surgery, multidisciplinary approach not encouraged, opioids routinely used, bladder catheter routinely used, ICU support mandatory, functional rehabilitation commenced on the first or second postoperative day. No functional discharge criteria	3 months
den Hartog et al. 2013 [55]	Netherlands	THR	Short-stay: 384 Usual care: 157	Short-stay: 69 Usual care: 68	Short-stay: 71 (10) Usual care: 71 (10)	- Mean LoS 2.9 days - 10 evidence-based interventions across the pre-operative, intraoperative, and postoperative period	- Mean LoS 4.6 days - Functional discharge criteria, sufficient pain treatment required before discharge (encompassing 2/10 interventions)	3 months
Dhawan et al. 2017 [26]	England	TKR	Short-stay: 50 Usual care: 70	Short-stay: 42 Usual care: 40	Median [range] Female: 70 [42–90] Male: 72 [55-81]	- LoS reduced by 1.5 days in both males and females - Local anaesthetic, tourniquets released before closure, homeostasis obtained, no drains used	- LoS not reported - No usual care components reported	During admission
Didden et al. 2019 [56]	Netherlands	TKR	Short-stay: 85 Usual care: 85	Short-stay: 60 Usual care: 64	Mean [range] Short-stay: 69 [52–86] Usual care: 69 [47–86]	- Median LoS 4 days - Local infiltration analgesia, oxycodone as needed only, mobilisation started 4 h after surgery, targeted discharge as soon as possible after surgery	- Median LoS 7 days - Epidural analgesia or femoral nerve block, prolonged-release oxycodone for a maximum of 10 days post-discharge, mobilisation started the day after surgery, targeted discharge 4 days after surgery	90 days
Doman et al. 2012 [39]	United States	THR (39) TKR (61)	Short-stay: 90 Usual care: 85	Short-stay: 38 Usual care: 58	Short-stay: 62 Usual care: 59	- Mean LoS 2.6 days - Patient education was used to guide recovery expectations, preoperative pain medication was initiated the morning of surgery, IV sedation was encouraged, efforts were made to use the minimal incision length and early mobilisation	- Mean LoS 5.5 days - No preoperative analgesia, the surgical technique did not emphasise smaller incisions, no established pain regimen, patients discharged once the pain was controlled via oral medication	30 days
Dwyer et al. 2012 [37]	United Kingdom	THR	Short-stay: 64 Usual care: 63	Short-stay: 58 Usual care: 65	Short-stay: 70.1 (8.8) Usual care: 72.5 (8.7)	- Mean LoS 5.3 days - Preadmission, preoperative, intraoperative, and postoperative interventions but with a focus on preoperative and postoperative nutrition	- Mean LoS 8.3 days - Usual care pathway not reported	Not reported, but outcomes are provided up to 81 days postop
Dwyer et al. 2014 [27]	United Kingdom	TKR	Short-stay: 57 Usual care: 55	Short-stay: 70 Usual care: 60	Short-stay: 71 (9) Usual care: 73 (9)	- Mean LoS 6 days - Preadmission interventions of multidisciplinary and holistic care and family involvement, and 10 evidence-based interventions across the pre-operative, intraoperative, and postoperative period	- Mean LoS 7.8 days - Patients admitted the evening before surgery, spinal or general anaesthesia used, mobilisation not started until the day after surgery, no routine physiotherapy	3 weeks
Featherall et al. 2018 [40]	United States	THR	Short-stay: 2,081 Usual care: 1,033	Short-stay: 50 Usual care: 50	Short-stay: 64 (12) Usual care: 64 (12)	- Mean LoS 2.55 days - Preoperative assessment and risk factor modification, antibiotic prophylaxis, tranexamic acid, operating room adjustments, and standardised postoperative care	- Mean LoS 3.21 days - Usual care pathway not described	90 days
Galbraith et al. 2017 [70]	Ireland	THR (40) TKR (60)	Short-stay: 165 Usual care: 145	Short-stay: 42 Usual care: 48	Not reported	- Mean LoS 5.1 days - Multidisciplinary care, optimisation of pre-existing conditions, drains not used, local infiltration analgesia, tranexamic acid, early mobilisation	- Mean LoS 8.79 days - Admitted the day before surgery, spinal or general anaesthesia used, analgesics provided, opioids commonly used for pain, mobilisation 1-day post-surgery	90 days
Gleicher et al. 2021 [58]	Canada	TKR	Short-stay: 383 Usual care: 232	Short-stay: 60 Usual care: 64	Short-stay: 67 (10) Usual care: 66 (10)	- Mean LoS 2.13 days - Postoperative analgesia, nausea and vomiting prophylaxis, Foley catheterisation, patient education	- Mean LoS 2.81 days - Perioperative placement of adductor canal block, IV dexamethasone, avoid unnecessary Foley catheterization, patient education not a focus	30 days
Gwynne-Jones 2017 [62]	New Zealand	THR (61) TKR (39)	Short-stay: 528 Usual care: 507	Short-stay: 54 Usual care: 54	Short-stay THR: 68 (12) Usual care THR: 67 (12) Short-stay TKR: 70 (9) Usual care TKR: 70 (9)	- Mean LoS THR 4.3 days - Mean LoS TKR 4.8 days - Optimisation of pre-existing conditions, patient education, standardised anaesthesia and analgesia, blood management, early mobilisation	- Mean LoS THR 5.6 days - Mean LoS TKR 5.7 days - Usual care pathway not described	90 days
Harkouk et al. 2021 [49]	France	THR (13) TKR (27)	Short-stay: 203 Usual care: 294	Short-stay: 66 Usual care: 67	Short-stay THR: 66 (14) Usual care THR: 68 (12) Short-stay TKR: 68 (12) Usual care TKR: 71 (11)	- Mean LoS THR 8.2 days - Mean LoS TKR 7.1 days - Staff education	- Mean LoS THR 8.2 days - Mean LoS TKR 8.7 days - No staff education	30 days
Joo et al. 2022 [66]	Australia	THR (34) TKR (66)	Short-stay: 146 Usual care: 143	Short-stay: 49 Usual care: 50	Short-stay: 69 (9) Usual care: 69 (9)	- Mean LoS 2.29 days - Early mobilisation, functional discharge criteria	- Mean LoS 3.24 days - Mobilisation the day post-surgery, no functional discharge criteria	3 months
Khan et al. 2014 [28]	United Kingdom	THR (42) TKR (58)	Short-stay: 3000 Usual care: 3000	Short-stay: 54 Usual care: 51	Short-stay: 68 (10) Usual care: 69 (10)	- Mean LoS 3 days - Pharmacological (low-dose spinal anaesthesia), procedural (early mobilisation), and behavioural (Patient and staff education) interventions	- Mean LoS 6 days - Pharmacological (general anaesthesia), procedural (catheterisation and next-day mobilisation), and behavioural (generic patient and staff education) interventions	90 days
Larsen et al. 2008 [60]	Denmark	THR TKR	Short-stay: 142 Usual care: 105	Not reported	No reported	- Mean LoS 4.4 days - Preoperative assessment and information, nutrition optimisation, early mobilisation	- Mean LoS 8.8 days - Patients received identical operation procedures, pain relief medication, and nausea controls	90 days
Maempel et al. 2015 [30]	United Kingdom	TKR	Short-stay: 84 Usual care: 81	Short-stay: 50 Usual care: 54	Short-stay: 70 (9) Usual care: 70 (11)	- Mean LoS 3 days - Patient education, spinal anaesthesia, tranexamic acid, early mobilisation	- Mean LoS 4 days - Spinal anaesthesia, tranexamic acid, patient-controlled analgesia	During admission
Maempel et al. 2016 [29]	United Kingdom	THR	Short-stay: 550 Usual care: 611	Short-stay: 62 Usual care: 60	Short-stay: 64 [16] Usual care: 66 [15]	- LoS reduced by a mean of 1.5 days - Patient education, functional discharge criteria, spine anaesthetic, early mobilisation	- LoS not reported - Post-controlled analgesia, mobilisation 1-day post-surgery, a spine anaesthetic,	During admission
Malviya et al. 2011 [31]	United Kingdom	THR (42) TKR (58)	Short-stay: 1500 Usual care: 3000	Short-stay: 53 Usual care: 51	Short-stay: 68 Usual care: 69	- Mean LoS 3 days - Targeted patient and staff education, low-dose spinal anaesthesia, tranexamic acid, local anaesthetic, early mobilisation, functional discharge criteria	- Mean LoS 6 days - Generic patient and staff education, general anaesthesia, catheterisation, mobilisation 1-day post-surgery, patient-controlled analgesia	60 days
McDonald 2012 [32]	United Kingdom	TKR	Short-stay: 1081 Usual care: 735	Short-stay: 59 Usual care: 42	Short-stay: 69 (11) Usual care: 71 (13)	- Mean LoS 4 days - Patient education, perioperative multimodal analgesia, local intra-articular injection, and early mobilisation	- Mean LoS 6 days - Tranexamic acid, spinal anaesthesia, mobilisation 1-day post-surgery	90 days
Picart et al. 2021 [50]	France	TKR	Short-stay: 216 Usual care: 335	Short-stay: 63 Usual care: 66	Short-stay: 69 (8) Usual care: 69 (10)	- Mean LoS 6.12 days - Patient education, no perineural block, tourniquet, or drainage, tranexamic acid, early mobilisation	- Mean LoS 6.30 days - Surgery under perineural block and tourniquet, post-operative drainage	90 days
Raphael et al. 2011 [59]	Canada	THR (57) TKR (43)	Short-stay: 100 Usual care: 100	Short-stay: 48 Usual care: 53	Short-stay: 65 (9) Usual care: 69 (8)	- Mean LoS 47 h - Patient education, pre-emptive analgesia, patient-controlled opioid analgesia, early mobilisation, and functional discharge criteria	- Mean LoS 116 h - Limited patient education, no standardised pre or post-operative multimodal analgesia, use of peripheral nerve block for postoperative analgesia mobilisation 1-day post-surgery	30 days
Romano et al. 2021 [53]	Italy	THR (52) TKR (48)	Short-stay: 122 Usual care: 59	Short-stay: 45 Usual care: 53	Short-stay: 70 [64-77] Usual care: 73 [68-77]	- Median LoS 5 days - Preadmission care, preoperative and intraoperative care, and postoperative care with a focus on patient education, anaesthesia and pain control, wound management and early mobilisation	- Median LoS 8 days - No patient education, no standard protocol for oral analgesia, conventional anaesthesia and sedation, no prevention of postoperative nausea and vomiting, mobilisation 1-day post-surgery	1 month
Savaridas et al. 2013 [34]	United Kingdom	THR (42) TKR (58)	Short-stay: 1,500 Usual care: 3,000	Short-stay: 53 Usual care: 51	Short-stay: 68 Usual care: 69	- Length of stay not reported - Targeted patient and staff education, low-dose spinal anaesthesia, tranexamic acid, local anaesthetic, early mobilisation, functional discharge criteria	- Length of stay not reported - Generic patient and staff education, general anaesthesia, catheterisation, mobilisation 1-day post-surgery, patient-controlled analgesia	3 months
Stambough et al. 2015 [41]	United States	THR	Short-stay: 488 Usual care: 281	Short-stay: 49 Usual care: 55	Short-stay: 55 [45-64] Usual care: 59 [51-67]	- Median LoS 2 days - Five targets of patient education (mandatory), pain (intra-op, no opioids), mobilisation (early mobilisation), anaesthesia (patient-specific spinal), and nursing (staff coordination)	- Median LoS 4 days - No patient education, patient-controlled analgesia, mobilisation delayed, general anaesthesia, and nursing not integrated into postoperative care	30 days
Starks et al. 2014 [35]	England	THR (41) TKR (59)	Short-stay: 2,128 Usual care: 2,065	Short-stay: 65 Usual care: 66	Mean [range] Short-stay: 71 [28–93] Usual care: 72 [26–98]	- Median LoS TKR 4 days - Median LoS THR 4 days - Patient education, spinal anaesthetic, normothermia, standardised analgesic ladder, and early mobilisation	- Median LoS TKR 6 days - Median LoS THR 6 days - Usual care pathway not reported	30 days
Stowers et al. 2016 [63]	New Zealand	THR (31) TKR (69)	Short-stay: 100 Usual care: 100	Short-stay: 53 Usual care: 59	Short-stay: 67 (9) Usual care: 65 (13)	- Mean LoS 4 days - Short-stay pathway details located in supplementary material are no longer accessible	- Mean LoS 5 days - Usual care pathway details located in supplementary material are no longer accessible	30 days
Tasso et al. 2022 [54]	Italy	THR (69) TKR (31)	Short-stay: 2,806 Usual care: 2,236	Short-stay: 57 Usual care: 56	Short-stay: 69 (7) Usual care: 67 (8)	- Mean LoS 5.1 days - Pre-admission evaluation, hospital admission, surgical strategies, anaesthesia, blood management, and early mobilisation	- Mean LoS 10.4 days - Usual care pathway not reported	30 days
Taylor et al. 2022 [42]	United States	THR (33) TKR (31)	Short-stay: 279 Usual care: 294	Short-stay: 67 Usual care: 66	Short-stay: 61 (10) Usual care: 61 (10)	- Mean LoS 1.6 days - Patient education, anaesthesia regimen and surgical protocol, early mobilisation, and multimodal pain control	- Mean LoS 3.0 days - Usual care pathway not reported	90 days
Teeny et al. 2005 [43]	United States	TKR	Short-stay: 55 Usual care: 55	Short-stay: 69 Usual care: 71	Mean [range] Short-stay: 70 [42–86] Usual care: 69 [41–84]	- Mean LoS 4.4 days - Intravenous fluids discontinued 1-day postop, catheters in place for a maximum of 24 h, early mobilisation	- Mean LoS 5.7 days - Intravenous fluids discontinued 2 days postop, catheters in place for up to 48 h, mobilisation the day after surgery	3 months
Yanik et al. 2018 [44]	United States	THR (30) TKR (70)	Short-stay: 78 Usual care: 174	Short-stay: 10 Usual care: 10	Short-stay: 66 (9) Usual care: 66 (9)	- Mean LoS 1.7 days - Patient education, spinal anaesthesia without femoral nerve blocks, multimodal pain techniques, and early mobilisation	- Mean LoS 3.2 days - Usual care pathway not reported	90 days
**Other observational study designs**								
Castorina et al. 2017 [52] Retrospective observational study	Italy	TKR	Short-stay: 95 Usual care: 37	Not reported	Short-stay: 71 (7) Usual care: 74 (6)	- LoS not reported - No tourniquet used, tranexamic acid used at 3-time points, no articular drainage	- LoS not reported - Regular articular drainage was used, tranexamic acid was only used at two time points	2 months
Edelmann et al. 2022 [73] Retrospective observational study	Switzerland	THR (62) TKR (38)	Short-stay: 302 Usual care: 138	Short-stay THR: 51 Usual care THR: 42 Short-stay and usual care TKR: 64	Short-stay THR: 61 (11) Usual care THR: 67 (11) Short-stay TKR: 64 (11) Usual care TKR: 67 (9)	- Mean LoS TKR 6.0 days - Mean LoS THR 5.3 days - Preoperative education and counselling, preoperative physiotherapy, local anaesthetic for infiltration and nerve blocks, perioperative oral analgesia, early mobilisation, continuous audit and improvement	- Mean LoS TKR 8.4 days - Mean LoS THR 7.7 days - No patient education, no preoperative physiotherapy, no local anaesthetic, no oral analgesia, no early mobilisation, no continuous improvement and audit	90 days
Jiang et al. 2022 [46] Non-randomised prospective controlled study	China	TKR	Short-stay: 106 Usual care: 142	Short-stay: 55 Usual care: 59	Short-stay: 74 (6) Usual care: 75 (6)	- Mean LoS 9.6 days - Preoperative, intraoperative, and postoperative interventions with a focus on patient education, preoperative analgesia, and fasting guidelines	- Mean LoS 11.3 days - General anaesthetic, no multimodal analgesia, no blood protocol, mobilisation 1-day post-surgery	5 days
Liao et al. 2022 [47] Non-randomised controlled study (retrospective allocation to control and intervention groups	China	TKR	Short-stay: 40 Usual care: 40	Short-stay: 60 Usual care: 55	Short-stay: 65 (5) Usual care: 65 (5)	- Length of stay not reported - General anaesthetic, operation room temperature adjusted to a lower temperature, all fluids headed to a specific temperature, body temperature monitored, thermal mattress used	- LoS not reported - No special heat preservation method was adopted during the surgery	3 months
Reinhard et al. 2023 [68] Retrospective matched pair analysis	Germany	THR	Short-stay: 315 Usual care: 315	Short-stay: 43 Usual care: 43	Short-stay: 65 [20] Usual care: 65 [20]	- Length of stay not reported - Local infiltration analgesia, patient education, gain training, tranexamic acid, local infiltration analgesia, early mobilisation	- Length of stay not reported - No gait training or analgesic medication before surgery, long-lasting spinal anaesthesia, no tranexamic acid, drains applied	24 h
Ripolles-Melchor et al. 2019 [72] Non-randomised prospective controlled study (allocated to control and intervention groups based on hospital)	Spain	THR (37) TKR (63)	Short-stay: 1592 Usual care: 4554	Short-stay: 58 Usual care: 58	Short-stay: 70 [63–76] Usual care: 71 [64–76]	- Median LoS 4 days - Sixteen Enhanced Recovery After Surgery Components based on Soffin & YaDeau’s recommendations	- Mean LoS 5 days - Usual care pathway not defined; individual patients were allocated to intervention or control groups based on the hospital’s short-stay protocols	30 days
Scott et al. 2012 [36] Consecutive snapshot audits from all 22 orthopaedic units in Scotland	Scotland	THR (52) TKR (48)	Short-stay: 405 Usual care: 873	Not reported	Short-stay: 68 (11) Usual care: 68 (10)	- Median LoS 4 days - Optimisation of pre-existing conditions, patient education, non-opioid multimodal analgesia, early mobilisation, and functional discharge	- Median LoS 5 days - Usual care pathway not reported	12 weeks
Slim et al. 2022 [51] Non-randomised matched groups (retrospective allocation to control and intervention groups)	France	THR TKR	Short-stay: 21,081 Usual care: 21,081	Not reported	Not reported	- LoS not reported - Short-stay pathway not described, but episodes of short-stay care were coded for inclusion in the study	- LoS not reported - Usual care pathway not reported	90 days
Wang et al. 2023 [48]	China	THR	Short-stay: 45 Usual care: 45	Short-stay: 71 Usual care: 56	Short-stay: 66 (9) Usual care: 74 (12)	- Mean LoS 14 days - Preoperative optimisation, patient education, preoperative nutrition, multimodal pain relief, controlling body temperature, and early mobilisation	- Mean LoS 16 days - No protocol for pre-operative management	3 days

*IQR* interquartile range, *LoS* length of stay, *SD* standard deviation, *THR* total hip replacement, *TKR* total knee replacement

Three (6%) included studies were RCTs [33, 57, 61] and the remainder (*n* = 46, 94%) used a quasi-experimental study design [25–32, 34–56, 58–60, 62–73]. Most had an interrupted time series design (*n* = 35, 76%) [25–32, 34, 35, 37–45, 49, 50, 53–56, 58–60, 62, 63, 66, 67, 69–71] where post-implementation data were compared with pre-implementation data. Nine (20%) used other observational designs [36, 46–48, 51, 52, 68, 72, 73], and two were registry data studies (4%) [64, 65].

More than half (*n* = 25, 51%) included participants undergoing either hip or KR [25, 28, 31, 34–36, 38, 39, 42, 44, 45, 49, 51, 53, 54, 59, 60, 62–66, 70, 72, 73], 13 (27%) included participants undergoing TKR only [26, 27, 30, 32, 43, 46, 47, 50, 52, 56–58, 69], 11 (22%) included participants undergoing THR only [11, 29, 37, 40, 41, 48, 55, 61, 67, 68, 71], one included participants undergoing unicompartmental KR [33], one included participants undergoing bilateral total KR [69] and one included participants undergoing either unilateral or same-day bilateral THR [67].

The sample size varied substantially between studies, ranging from 41 participants in an RCT [33] to 116,293 participants in an arthroplasty registry-based study [64]. Females represented the majority (*n* = 31, 63%) of short-stay joint replacement participants in most studies [27–32, 34, 35, 37, 40, 42, 43, 45–50, 54, 55, 57, 58, 62–65, 67, 69, 71–73] and participant sex was not provided in four studies [36, 51, 52, 60]. Among the studies that reported age, participants were aged between ≤ 49 and 90 years with age not provided in three studies [51, 60, 70].

### Intervention characteristics

Short-stay interventions varied considerably across studies, in both their scope and content, and how they were described. As shown in Table 2, the most common short-stay interventions were early mobilisation (*n* = 41, 84%), perioperative information (*n* = 37, 76%), perioperative oral analgesia (*n* = 35, 71%), use of local anaesthesia for infiltration analgesia and nerve blocks (*n* = 34, 69%) and criteria-based discharge (*n* = 31, 63%).

**Table 2:** Short-Stay Programme Components Linked to ERAS Society Recommendations

	**Perioperative information**	**Preoperative optimization**	**Preoperative fasting**	**Standard anaesthetic protocol**	**Use of local anaesthetics for infiltration analgesia and nerve blocks**	**Postoperative nausea and vomiting prevention**	**Prevention of perioperative blood loss**	**Perioperative oral analgesia**	**Maintaining normothermia**
Study									
	Randomised Controlled Trials								
Fransen					✓			✓	
Petersen	✓			✓					
Reilly	✓								
	Registry Studies								
Berg 2018 [65]	✓				✓		✓	✓	
Berg 2021 [64]	✓				✓			✓	
	Interrupted Time Series Studies								
Alvis						✓	✓	✓	
Amlie					✓			✓	
Arshad	✓			✓	✓			✓	
Azam	✓	✓	✓	✓	✓	✓	✓	✓	✓
Chung					✓	✓	✓	✓	
de Carvalho Almeida	✓				✓		✓	✓	
den Hartog	✓				✓			✓	
Dhawan					✓		✓		
Didden	✓				✓			✓	
Doman	✓				✓			✓	
Dwyer 2012 [37]	✓	✓	✓		✓	✓		✓	
Dwyer 2014 [27]	✓		✓		✓	✓	✓	✓	
Featherall	✓	✓					✓	✓	
Galbraith	✓				✓		✓	✓	
Gleicher	✓					✓		✓	
Gwynne-Jones		✓		✓	✓		✓	✓	✓
Harkouk	✓								
Joo	✓								
Khan	✓			✓	✓		✓	✓	
Larsen	✓					✓			
Maempel 2015 [30]	✓				✓		✓		
Maempel 2016 [29]	✓				✓				
Malviya	✓				✓		✓	✓	
McDonald	✓				✓	✓	✓	✓	
Picart	✓	✓	✓		✓		✓		
Raphael	✓				✓	✓		✓	
Romano	✓	✓				✓	✓	✓	✓
Savaridas	✓				✓		✓	✓	
Stambough	✓				✓			✓	
Starks	✓	✓			✓			✓	✓
Stowers									
Tasso		✓			✓			✓	✓
Taylor	✓	✓			✓			✓	
Teeny	✓								
Yanik	✓				✓			✓	✓
	Other Observational Study Designs								
Castorina							✓	✓	
Edelmann	✓				✓			✓	
Jiang			✓		✓	✓	✓	✓	
Liao	✓		✓					✓	✓
Reinhard	✓				✓		✓		
Ripolles-Melchor	✓	✓	✓			✓		✓	✓
Scott	✓		✓		✓				
Slim									
Wang	✓				✓				
**Total**	**37**	**10**	**8**	**5**	**34**	**12**	**20**	**35**	**8**

	**Antimicrobial prophylaxis**	**Antithrombotic prophylaxis treatment**	**Perioperative surgical factors**	**Perioperative fluid management**	**Postoperative nutrition care**	**Early mobilisation**	**Criteria-based discharge**	**Continuous improvement and audit**
Study								
Fransen		✓	✓			✓	✓	
Petersen			✓		✓	✓	✓	
Reilly		✓				✓	✓	✓
Berg 2018 [65]		✓	✓			✓	✓	
Berg 2021 [64]						✓	✓	
Alvis				✓	✓	✓	✓	
Amlie						✓		
Arshad			✓			✓		
Azam	✓	✓	✓	✓	✓	✓	✓	
Chung		✓	✓			✓		
de Carvalho Almeida	✓		✓			✓	✓	
den Hartog			✓			✓	✓	
Dhawan			✓					
Didden						✓	✓	
Doman			✓			✓	✓	
Dwyer 2012 [37]					✓	✓	✓	
Dwyer 2014 [27]			✓	✓	✓	✓	✓	
Featherall	✓	✓				✓	✓	
Galbraith			✓			✓	✓	
Gleicher			✓			✓	✓	
Gwynne-Jones	✓			✓		✓	✓	
Harkouk								
Joo						✓	✓	
Khan		✓	✓	✓		✓		
Larsen						✓		✓
Maempel 2015 [30]				✓			✓	
Maempel 2016 [29]			✓			✓	✓	
Malviya		✓		✓		✓	✓	
McDonald			✓	✓		✓	✓	
Picart							✓	
Raphael		✓	✓			✓	✓	
Romano			✓	✓	✓	✓		
Savaridas				✓		✓	✓	
Stambough						✓	✓	
Starks	✓		✓				✓	
Stowers			✓					
Tasso			✓			✓		
Taylor						✓	✓	
Teeny		✓	✓			✓		
Yanik		✓	✓		✓	✓	✓	
Castorina			✓			✓		
Edelmann						✓		
Jiang				✓		✓		
Liao					✓	✓		
Reinhard			✓			✓		
Ripolles-Melchor	✓			✓	✓	✓		
Scott						✓	✓	
Slim								
Wang								
**Total**	**6**	**11**	**25**	**12**	**9**	**41**	**31**	**2**

Additional short-stay components that were used in the included studies, but are not a part of the ERAS Society recommendations, include (1) patient admission the night before or the morning of surgery [29, 30, 35, 39, 50, 54, 56, 58, 60, 64, 65, 69, 70], (2) multidisciplinary staff (for example, physiotherapists, occupational therapists, social workers) working with short-stay patients for holistic care [27, 32, 38, 40, 41, 47, 53, 54, 58, 67, 70], (3) preoperative carbohydrate and/or protein loading to reduce the metabolic stress of starvation [27, 46–48, 60, 69, 72], (4) preoperative staff education on short-stay joint replacement programmes [28, 31, 34, 49, 58, 60, 69], (5) hypnotics to promote patient compliance with early mobilisation [45], (6) wearing patients’ own clothes during admission to promote patient comfort and satisfaction [43, 60], (7) not using negative vacuum suction drains [69, 71], [8] low tidal volume ventilation strategy to prevent ventilator-associated lung injury [38, 62], (9) higher dose of steroids [45], and (11) preoperative physiotherapy [73].

### Risk of bias assessment results

#### Randomised controlled trials

Risk of bias results for the RCTs can be found in Supplementary File 3. All three trials were at low risk of selection bias but they were all at high risk of performance and detection bias as allocation to treatment groups was not concealed and neither participants nor the treating surgeons were blinded. One trial blinded the surgeon responsible for discharging participants [61], one blinded the physiotherapist responsible for collecting patient-reported outcome data (this occurred at 6 months and was not included in our review) [33], and one did not attempt to blind staff [57]. Two studies were at low risk of assessment bias [33, 61], but one was at high risk based on unclear information on participants lost to follow-up [57]. There were few losses to follow-up, reflecting a low risk of attrition bias. Appropriate statistical analysis was used in all trials.

#### Quasi-experimental studies

Risk of bias results for the quasi-experimental studies can be found in Supplementary File 3. The temporal relationship of the variables was clear in all but one study [51]. The two registry studies were at low risk of selection bias as participants were from the Swedish Hip and Knee Arthroplasty Registers, which have 100% national coverage and 96–98% completeness for primary THR and TKR surgeries [64, 65]. All other quasi-experimental studies were at high risk of selection bias: 22 studies due to between-group differences at baseline that may have influenced study outcomes [25, 26, 28, 29, 31, 34–37, 39–41, 44, 48, 49, 52, 59, 64, 65, 71–73] and one due to unclear descriptions of the short-stay intervention compared with usual care [55]. Twenty-four studies provided data showing that short-stay and usual care group participants had comparable demographics [27, 30, 32, 38, 42, 43, 45–47, 50, 51, 53–56, 58, 60, 62, 66–70].

Only ten studies conducted multiple outcome assessments both pre- and post-intervention [29–32, 43, 46–48, 54, 65], but most outcomes included in this review only required one measurement (for example, readmission, mortality, reoperation).

Both registry studies were judged to be at high risk of loss to follow-up due to unclear explanations of participants who were potentially lost to follow-up within the registry [64, 65]. All other quasi-experimental studies were at high risk of loss to follow-up, but 25 provided data on the number of participants who did not complete the study [25, 27, 30, 32, 34, 35, 38–42, 44, 45, 47, 49–51, 53, 55, 58, 60, 62, 67, 69, 73]. We found that 20 studies were at risk of lacking validity based on outcomes not being measured in a reliable way, or the use of inappropriate statistical analysis [30, 35–37, 44–46, 48, 51, 52, 54, 55, 58, 62, 63, 66–70]. All studies were considered at low risk of reporting bias.

### Effects of interventions

The safety outcomes included in each study can be found in Table 3.

**Table 3:** Outcomes included in each study

Study	Readmissions	Reoperation	Blood Loss (including Requires Blood Transfusion)	ED Visits	Infection	Mortality	Neurovascular Injury	Other Complications	Periprosthetic Fractures	Postoperative Falls	Venous Thromboembolism	Wound Complications	Dislocation	Stiffness and/or including Manipulation
	Randomised Controlled Trials													
Fransen 2018 [57]							✓	✓						✓
Petersen 2006 [61]	✓		✓					✓						
Reilly 2005 [33]	✓							✓						✓
	Registry Studies													
Berg 2018 [65]	✓				✓	✓	✓	✓			✓	✓	✓	✓
Berg 2021 [64]						✓						✓		
	Interrupted Time Series Studies													
Alvis 2021 [38]	✓													
Amlie 2016 [71]		✓												
Arshad 2014 [25]		✓	✓					✓				✓		
Azam 2022 [69]	✓		✓		✓	✓		✓			✓			
Chung 2021 [45]	✓				✓			✓	✓	✓			✓	
de Carvalho Almeida 2021 [67]	✓		✓		✓	✓	✓	✓	✓				✓	
den Hartog 2013 [55]	✓				✓			✓					✓	
Dhawan 2017 [26]														
Didden 2019 [56]	✓													
Doman 2012 [39]	✓													
Dwyer 2012 [37]	✓							✓			✓		✓	
Dwyer 2014 [27]								✓						
Featherall 2018 [40]								✓						
Galbraith 2017 [70]	✓													
Gleicher 2021 [58]				✓				✓						
Gwynne-Jones 2017 [62]	✓		✓			✓						✓		
Harkouk 2021 [49]	✓							✓						
Joo 2022 [66]	✓							✓						
Khan 2014 [28]	✓	✓	✓			✓		✓			✓			
Larsen 2008 [60]						✓		✓						
Maempel 2015 [30]			✓					✓				✓		
Maempel 2016 [29]								✓						
Malviya 2011 [31]	✓	✓	✓			✓		✓			✓			
McDonald 2012 [32]			✓					✓			✓			
Picart 2021 [50]		✓	✓		✓	✓		✓			✓			✓
Raphael 2011 [59]	✓		✓	✓										
Romano 2021 [53]			✓					✓						
Savaridas 2013 [34]						✓								
Stambough 2015 [41]	✓													
Starks 2014 [35]	✓					✓								
Stowers 2016 [63]	✓	✓			✓	✓		✓				✓	✓	
Tasso 2022 [54]	✓	✓	✓			✓					✓			
Taylor 2022 [42]	✓	✓	✓					✓			✓	✓		
Teeny 2005 [43]								✓			✓	✓		✓
Yanik 2018 [44]	✓	✓						✓				✓		
	Other Observational Study Designs													
Castorina 2017 [52]			✓					✓						
Edelmann 2022 [73]								✓						
Jiang 2019 [46]						✓								
Liao 2022 [47]					✓			✓			✓			
Reinhard 2023 [68]								✓						
Ripolles-Melchor 2019 [72]	✓		✓		✓	✓		✓			✓	✓		
Scott 2012 [36]			✓					✓						
Slim 2022 [51]						✓								
Wang 2023 [48]			✓											
**Total**	**25**	**9**	**18**	**2**	**9**	**16**	**3**	**33**	**2**	**1**	**12**	**10**	**6**	**5**

#### RCT evidence

Data from the RCTs were available for only five of the pre-specified 14 safety outcomes: readmissions [33, 61], blood loss (including requiring blood transfusion) [61], other complications [33, 57, 61], neurovascular injury [57], and stiffness and/or manipulation [33, 57]. Table 4 displays the GRADE results, Supplementary File 4 displays the forest plots, and Supplementary File 5 summarises reported outcome data that were not able to be included in pooled analyses.

**Table 4:** Assessment of Evidence Certainty using GRADE

Summary of findings:						
**Short-stay compared to usual care for total hip and knee replacement**						
**Patient or population:** Adults ≥ 18 years undergoing elective THR or knee replacement (unilateral, bilateral, total, unicompartmental) **Setting:** Any setting that utilised a short-stay programme **Intervention:** Short-stay **Comparison:** Usual care						
Outcome № of participants (studies)	Relative effect (95% CI)	**Anticipated absolute effects (95% CI)**			Certainty	What happens
		**Without Short-Stay**	**With Short-Stay**	**Difference**		
Blood transfusion № of participants: 57 (1 RCT)	**OR 1.75** (0.27 to 11.36)	6.7%	**11.1%** (1.9 to 44.8)	**4.4% more** (4.8 fewer to 38.1 more)	⨁⨁◯◯ Low^a,b^	Short-stay programmes may result in little to no difference in blood transfusion
Other Complications № of participants: 147 (3 RCTs)	**OR 0.63** (0.26 to 1.53)	23.0%	**15.8%** (7.2 to 31.3)	**7.2% fewer** (15.8 fewer to 8.4 more)	⨁⨁◯◯ Low^b^	Short-stay programmes may result in little to no difference in other complications
Hospital Readmissions № of participants: 98 (2 RCTs)	**OR 0.95** (0.12 to 7.46)	4.0%	**3.8%** (0.5 to 23.7)	**0.2% fewer** (3.5 fewer to 19.7 more)	⨁⨁◯◯ Low^b^	Short-stay programmes may result in little to no difference in hospital readmissions
Stiffness and/or anipulation № of participants: 90 (2 RCTs)	**OR 1.57** (0.18 to 13.26)	2.3%	**3.5%** (0.4 to 23.6)	**1.2% more** (1.9 fewer to 21.3 more)	⨁⨁◯◯ Low^b^	Short-stay programmes may result in little to no difference in stiffness and/or manipulation
Neurovascular Injury № of participants: 49 (1 RCT)	**OR 0.31** (0.01 to 7.92)	4.2%	**1.3%** (0 to 25.6)	**2.8% fewer** (4.1 fewer to 21.4 more)	⨁⨁◯◯ Low^a,b^	Short-stay programmes may result in little to no difference in neurovascular injury
*The risk in the intervention group (and its 95% confidence interval) is based on the assumed risk in the comparison group and the **relative effect** of the intervention (and its 95% CI) *CI* confidence interval, *OR* odds ratio						
**GRADE Working Group grades of evidence** **High certainty:** we are very confident that the true effect lies close to that of the estimate of the effect **Moderate certainty:** we are moderately confident in the effect estimate: the true effect is likely to be close to the estimate of the effect, but there is a possibility that it is substantially different **Low certainty:** our confidence in the effect estimate is limited: the true effect may be substantially different from the estimate of the effect **Very low certainty:** we have very little confidence in the effect estimate: the true effect is likely to be substantially different from the estimate of effect						

Explanations: ^a^Small event rate from a single study, ^b^Although the RCTs were hampered by an inability to blind the interventions, this does not appear to bias the outcomes

##### Readmissions

There was low certainty evidence that short-stay programmes may not reduce the odds of hospital readmission compared to usual care (short-stay: 2/48 [4.2%], usual care: 2/50 [4.0%], OR 0.95, 95% CI 0.12 to 7.46; two trials, 98 participants). The certainty of evidence was downgraded for imprecision due to the small number of studies and events.

##### Blood transfusion

Compared with usual care, there was low certainty evidence that short-stay programmes may not reduce blood transfusion requirements (short-stay: 3/27 [11.1%], usual care: 2/30 [6.7%], OR 1.75, 95% CI 0.27 to 11.36; one trial, 57 participants). The certainty of evidence was downgraded for imprecision due to the very low event rate from a single study. There was less postoperative bleeding in the short-stay group (average 234.1 ml), compared to usual care (average 387.9 ml), but post-operative haemoglobin levels were similar (short-stay average 6.94, usual care average 6.94).

##### Neurovascular injury

There was low certainty evidence that short-stay programmes may not reduce the odds of neurovascular injury, compared to usual care (short-stay: 0/25 [0%], usual care: 1/24 [4.2%]; OR 0.31, 95% CI 0.01 to 7.92; one trial, 49 participants [57]). The certainty of the evidence was downgraded for imprecision due to the very low event rate from a single study.

##### Other complications

There was low certainty evidence that short-stay programmes may not reduce the odds of experiencing other complications, compared to usual care (short-stay: 11/73 [15.1%], usual care: 17/74 [23.0%]; OR 0.63, 95% CI 0.26 to 1.53; three trials, 147 participants, *I*^2^ = 0%). The certainty of the evidence was downgraded for imprecision due to the small number of studies and events.

##### Stiffness and/or manipulation

There was low certainty evidence that short-stay programmes may not reduce the odds of stiffness and/or requiring manipulation compared to usual care (short-stay: 2/46 [4.3%], usual care: 1/44 [2.3%]; OR 1.04, 95% CI 0.53 to 2.05; two trials, 90 participants, *I*^2^ = 0%). The certainty of evidence was downgraded for imprecision due to the small number of studies and events.

No trials assessed reoperations, emergency department visits, infection, mortality, periprosthetic fractures, postoperative falls, venous thromboembolism, wound complications, or dislocation.

#### Registry evidence

Data from the registries were available for nine of the pre-specified 14 safety outcomes: readmissions [65], infection [65], mortality [64, 65], neurovascular injury [65], other complications [65], venous thromboembolism [65], wound complications [65], dislocation [65], and stiffness and/or manipulation [65]. The certainty of the evidence was low. The evidence was not downgraded (there was no serious imprecision, no serious indirectness as the variability likely reflects what happens in practice, no inconsistency, and little evidence of publication bias) or upgraded (no large magnitude of effect and no evidence of a large dose–response gradient). Supplementary File 4 displays the forest plots and Supplementary File 5 summarises reported outcome data that were not able to be included in pooled analyses.

##### Infection

There was low certainty evidence that short-stay programmes may not reduce the odds of experiencing infection, compared to usual care (short-stay: 90/7345 [1.2%], usual care: 88/6803 [1.3%]; OR 0.95, 95% CI 0.70 to 1.27; one study; 14,148 participants).

##### Mortality

There was low certainty evidence that short-stay programmes may reduce the odds of mortality, compared to usual care (short-stay: 171/75,017 [0.2%], usual care: 195/55,424 [0.4%]; OR 0.64, 95% CI 0.52 to 0.79; two studies; 130,441 participants, *I*^2^ = 0%). The hazard ratios (HRs) of mortality within 30 and 90 days were lower in the fast-track group for both THR (HR 0.80, 95% CI 0.55 to 1.17) and TKR (HR 0.69, 95% CI 0.45 to 1.07).

##### Neurovascular injury

There was low certainty evidence that short-stay programmes may not reduce the odds of neurovascular injury, compared to usual care (short-stay: 28/7345 [0.4%], usual care: 26/6803 [0.4%]; OR 1.00, 95% CI 0.58 to 1.70; one study; 14,148 participants).

##### Other *complications*

There was low certainty evidence that short-stay programmes may not reduce the odds of experiencing other complications compared to usual care (short-stay: 563/7345 [7.7%], usual care: 511/6803 [7.5%], OR 1.03, 95% CI 0.90 to 1.16; one study, 14,148 participants). There was no difference in the odds of experiencing other complications between short-stay and usual care groups (short-stay THR 30 days: OR 1.1, 95% CI 0.9 to 1.3; short-stay THR 90 days: OR 1.1, 95% CI 0.9, 1.2; short-stay TKR 30 days: OR 1.1, 95% CI 0.9 to 1.3; short-stay TKR 90 days: OR 1.2, 95% CI 1.0 to 1.4).

##### Venous *thromboembolism*

There was low certainty evidence that short-stay programmes may not reduce the odds of venous thromboembolism, compared to usual care (short-stay: 80/7270 [1.1%], usual care: 67/6640 [1.0%], OR 1.09, 95% CI 0.79 to 1.51; one study, 13,910 participants).

##### Wound *complications*

There was low certainty evidence that short-stay programmes may not reduce the odds of wound complications, compared to usual care (short-stay: 84/7270 [1.2%], usual care: 90/6640 [1.4%], OR 0.85, 95% CI 0.63 to 1.15; one study; 13,910 participants).

##### Dislocation

There was low certainty evidence that short-stay programmes may reduce the odds of dislocation, compared to usual care (short-stay: 33/7345 [0.45%], usual care: 51/6803 [0.75]; OR 0.60, 95% CI 0.39 to 0.93; one study; 14,148 participants).

##### Stiffness and/or manipulation

There was low certainty evidence that short-stay programmes may not reduce the odds of stiffness and/or manipulation, compared to usual care (short-stay: 18/7345 [0.2%], usual care: 16/6803 [0.2%], OR 1.04, 95% CI 0.53 to 2.05; one study, 14,148 participants).

No registry studies assessed reoperations, blood loss (including requiring a blood transfusion), emergency department visits, periprosthetic fractures or postoperative falls.

#### Interrupted time series evidence

Data from the interrupted time series studies were available for 13 of the 14 pre-specified safety outcomes: readmissions [28, 31, 35, 37–39, 41, 42, 44, 45, 49, 50, 54–56, 59, 60, 62, 63, 66, 67, 69, 70], reoperations [25, 28, 31, 42, 44, 50, 54, 55, 63, 71], blood loss (including requiring a blood transfusion) [25, 28, 30–32, 42, 50, 53, 54, 59, 62, 67, 69], emergency department visits [58, 59], infection [45, 50, 55, 63, 67, 69], mortality [28, 31, 34, 35, 50, 54, 60, 62, 63, 67, 69], neurovascular injury [67], other complications [25, 27–32, 37, 40, 42–45, 49, 50, 53, 55, 58, 60, 63, 66, 67, 69], periprosthetic fracture [45, 67], venous thromboembolism [28, 31, 32, 37, 42, 43, 50, 54, 69], wound complications [25, 30, 42–45, 62, 63], dislocation [37, 45, 55, 63, 67] and stiffness and/or manipulation [43, 50].

The certainty of the evidence was low and not downgraded or upgraded. Supplementary File 4 displays the forest plots, and Supplementary File 5 summarises reported outcome data that were not able to be included in pooled analyses.

##### Readmissions

There was low certainty evidence that short-stay programmes may not reduce the odds of hospital readmissions, compared to usual care (short-stay: 443/12,571 [3.5%], usual care: 552/13,322 [4.1%]; OR 0.86, 95% CI 0.72 to 1.03; 21 studies; 25,893 participants; *I*^2^ = 18%). There was no significant difference in the percentage of readmissions from short-stay and usual care participants [55] and no significant difference in readmissions between short-stay and usual care groups at 30 and 90 days [70].

##### Reoperation

There was low certainty evidence that short-stay programmes may not reduce the odds of reoperation, compared to usual care (short-stay: 89/8266 [1.1%], usual care: 192/13,334 [1.4%]; OR 0.75, 95% CI 0.47 to 1.19; 9 studies; 21,600 participants; *I*^2^ = 48%).

##### Blood loss (including requiring a blood transfusion)

There was low certainty evidence that short-stay programmes may not reduce blood loss volume, compared to usual care (OR − 0.20, 95% CI − 0.98 to 0.59; two studies; 646 participants; *I*^2^ = 89%). There was low certainty evidence that short-stay programmes may reduce the odds of requiring a blood transfusion, compared to usual care (short-stay: 720/10,086 [0.7%], usual care: 1470/8631 [17.0%], OR 0.36, 95% CI 0.26 to 0.50; 13 studies, 18,717 participants; *I*^2^ = 82%).

Short-stay participants had a lower reduction in mean haemoglobin [30, 53, 69] and one study reported that this was significantly lower for the short-stay group [30]. Median postoperative haemoglobin levels were also significantly higher for short-stay participants (TKR short-stay: 11.5, usual care: 10.6, between group difference 0.02, 95% CI − 1.40, − 0.20; THR short-stay: 11.5, usual care: 10.1, between group difference > 0.01, 95% CI − 1.80 to − 0.60) [25]. Percentage blood loss was reported in two studies [31, 62] and found to be significantly lower for the short-stay group [31]. There was no between-group difference in the proportion of participants requiring intraoperative or postoperative transfusion in one study [59], but significantly lower for short-stay participants in two separate studies [50, 54].

##### Emergency department visits

There was low certainty evidence that short-stay programmes may not reduce the odds of emergency department visits, compared to usual care (short-stay 30/383 [7.8%], usual care 28/282 [9.9%]; OR 0.77, 95% CI 0.45 to 1.32; 2 studies; 665 participants; *I*^2^ = 0%).

##### Infection

There was low certainty evidence that short-stay programmes may not reduce the odds of infection, compared to usual care (short-stay: 11/1113 [0.99%], usual care: 9/950 [0.95%]; OR 0.77, 95% CI 0.29, 2.02; 6 studies; 2083 participants; *I*^2^ = 8%).

##### Mortality

There was low certainty evidence that short-stay programmes may not reduce the odds of mortality, compared to usual care (short-stay: 31/10,936 [0.28%], usual care: 77/9353 [0.82%]; OR 0.42, 95% CI 0.13, 1.35; 9 studies; 20,289 participants; *I*^2^ = 74%). Survival probability at 1 and 3 months was reported to be the same between short-stay and usual care participants [34] and the percentage of deaths was 0.1% for both groups in a separate study [54].

##### Neurovascular injury

There was low certainty evidence that short-stay programmes may not reduce the odds of neurovascular injury, compared to usual care (short-stay: 1/47 [2.1%], usual care: 2/51 [3.9%]; OR 0.53, 95% CI 0.05, 6.07; 1 study; 98 participants).

##### Other complications

There was low certainty evidence that short-stay programmes may reduce the odds of other complications, compared to usual care (short-stay: 953/10,621 [9.0%], usual care: 1306/11,743 [11.1%]; OR 0.71, 95% CI 0.58, 0.85; 22 studies; 22,364 participants; *I*^2^ = 63%). Two studies reported the percentage of other complications in the short-stay and usual care groups [55, 58] and one found a significantly reduced number of complications in the short-stay group [58]. Two studies reported the number of complications in the short-stay group only [25, 60].

##### Periprosthetic fracture

There was low certainty evidence that short-stay programmes may increase the odds of periprosthetic fracture, compared to usual care (short-stay: 4/158 [2.5%], usual care: 0/168 [0%], OR 5.25, 95% CI 0.59, 46.88; 2 studies; 326 participants; *I*^2^ = 0%).

##### Postoperative falls

Postoperative falls was the only pre-specified safety outcome that was unable to be pooled for analysis. One study reported on postoperative falls; it found one participant in the short-stay group had an accidental fall 13 days postoperatively and no falls were reported in the usual care group [45].

##### Venous thromboembolism

There was low certainty evidence that short-stay programmes may reduce the odds of venous thromboembolism, compared to usual care (short-stay: 87/9275 [0.9%], usual care: 148/9549 [1.5%]; OR 0.72, 95% CI 0.55 to 0.95; 9 studies 18,824 participants; *I*^2^ = 0%).

##### Wound complications

There was low certainty evidence that short-stay programmes may not reduce the odds of wound complications, compared to usual care (short-stay 41/1749 [2.3%], usual care: 36/1906 [1.9%]; OR 1.16, 95% CI 0.72 to 1.88; 8 studies; 3655 participants; *I*^2^ = 0%).

##### Dislocation

There was low certainty evidence that short-stay programmes may not reduce the odds of dislocation, compared to usual care (short-stay: 7/706 [1.0%], usual care: 5/488 [1.0%]; OR 1.02, 95% CI 0.33 to 3.18; 5 studies; 1,194 participants; *I*^2^ = 0%).

##### Stiffness and/or manipulation

There was low certainty evidence that short-stay programmes may reduce the odds of stiffness and/or manipulation, compared to usual care (short-stay: 2/271 [0.7%], usual care: 6/390 [1.5%]; OR 0.51, 95% CI 0.10 to 2.56; 2 studies; 661 participants).

#### Other observational study evidence

Data from the other observational studies were available for eight of the 14 pre-specified safety outcomes: readmissions [72], blood loss (including requiring a blood transfusion) [36, 48, 52], infection [47, 72], mortality [46, 51, 72], other complications [36, 47, 52, 72, 73], venous thromboembolism [47, 72] and wound complications [72]. The certainty of the evidence was low and not downgraded or upgraded. Supplementary File 4 displays the forest plots, and Supplementary File 5 summarises reported data that were not able to be included in the meta-analysis.

##### Readmissions

There was low certainty evidence that short-stay programmes may increase the odds of hospital readmission, compared to usual care (short-stay: 40/1592 [2.5%], usual care: 78/4554 [1.7%]; OR 1.48, 95% CI 1.01 to 2.17; one study; 118 participants).

##### Blood loss (including requiring a blood transfusion)

There was low certainty evidence that short-stay programmes may not reduce blood loss volume, compared to usual care (OR − 0.49, 95% CI − 1.15 to 0.17; one study; 132 participants). There was low certainty evidence that short-stay programmes may reduce the odds of requiring a blood transfusion, compared to usual care (short-stay: 24/500 [4.8%], usual care: 126/910 [13.8%]; OR 0.32, 95% CI 0.20 to 0.51; 2 studies; 1410 participants; *I*^2^ = 0%). One study reported significantly reduced postoperative haemorrhage in short-stay participants [72] and one study reported higher mean haemoglobin levels for the short-stay group at 1 and 3 days postoperatively, but did not adjust for higher preoperative haemoglobin levels in this group [48].

##### Infection

There was low certainty evidence that short-stay programmes may not reduce the odds of infection, compared to usual care (short-stay: 4/1632 [0.2%], usual care: 22/4594 [0.5%]; OR 0.39, 95% CI 0.13 to 1.15; 2 studies; 6226 participants; *I*^2^ = 8%).

##### Mortality

There was low certainty evidence that short-stay programmes may reduce the odds of mortality, compared to usual care (short-stay: 22/22,779 [0.1%], usual care: 42/25,776 [0.2%]; OR 0.57, 95% CI 0.34 to 0.95; 3 studies; 48,555 participants; *I*^2^ = 0%).

##### Other complications

There was low certainty evidence that short-stay programmes may not reduce the odds of other complications, compared to usual care (short-stay: 507/2434 [20.8%], usual care: 1110/5642 [19.7%]; OR 0.50, 95% CI 0.17 to 1.44; 5 studies; 8076 participants; *I*^2^ = 96%). One study found no significant difference in other complications between short-stay and usual care groups [68].

##### Venous thromboembolism

There was low certainty evidence that short-stay programmes may reduce the odds of venous thromboembolism, compared to usual care (short-stay: 7/1632 [0.4%], usual care: 38/4594 [0.8%]; OR 0.39, 95% CI 0.17 to 0.89; 2 studies; 6226 participants; *I*^2^ = 0%).

##### Wound complications

There was low certainty evidence that short-stay programmes may not reduce the odds of wound complications, compared to usual care (short-stay: 33/1592 [2.1%], usual care: 95/4554 [2.1%]; OR 0.99, 95% CI 0.67 to 1.48; one study; 6146 participants).

No observational studies assessed reoperations, emergency department visits, neurovascular injury, periprosthetic fracture, postoperative falls, dislocation, or stiffness and/or manipulation.

### Patient factors

Only one study reported data informing patient selection into short-stay programmes versus usual care [26]. This study reported comparable total blood loss for males and females in the short-stay group (*p* = 0.814), and comparable blood loss per unit body weight (mL/kg) for males and females in the short-stay group (*p* = 0.97). Four additional studies reported associations between patient factors and safety outcomes [40, 66, 71, 72], but these analyses included all study participants and were not specific to short-stay participants. None of the included studies examined relationships between patient factors and patient-reported pain, function, quality of life or satisfaction outcomes.

## Discussion

This systematic review evaluated the safety profile of short-stay programmes for people undergoing elective primary THR or KR, compared to usual care, across four study designs. We examined 14 safety outcomes up to 90 days post-operatively. Only five of these outcomes were included in RCTs, which demonstrated no evidence of harms with respect to hospital readmissions, blood transfusion requirements, other complications, neurovascular injury and stiffness and/or manipulation. However, due to the small number of trials and small number of participants this evidence is of low certainty and at best should be considered as evidence of non-inferiority.

While there is some evidence that short-stay joint replacement programmes are cost effective (saving up to $400 [USD] per patient) [14, 74], our review shows there are limited head-to-head comparisons with usual care that confirm their safety. The non-RCT studies (registry, interrupted time series and other observational studies) reported inconsistent findings and where benefits were observed (for example, for lower mortality, blood transfusion requirements, other complications, venous thromboembolism (VTE), dislocations and stiffness and/or manipulation), these results are likely to be overestimated, based on the smaller effect sizes seen with the RCT evidence for the same outcomes. Some safety outcomes have received relatively little attention to date. For example, only one study in our review examined postoperative falls despite an increased risk of this adverse event post-joint replacement surgery [75, 76]. Falls are an important but commonly overlooked safety outcome, given the potential for both in-hospital and post-discharge falls and sequalae that can include persistent disability or death [77].

We sought to review the evidence underpinning optimal patient selection; however, we identified only one study which reported data relevant to this aim (in relation to blood loss only). None of the included studies examined relationships between clinical or demographic factors and patient-reported pain, function, quality of life or satisfaction outcomes after surgery. This remains an important knowledge gap. One systematic review of patient-reported outcome measures in short-stay orthopaedic surgery in the UK showed that quality of life scores continued to improve up to 12 months post-operatively [78], but data on which patients achieve the greatest improvement is not available. A more recent study comparing short-stay and usual care joint replacement surgery in patients who have experienced both found that satisfaction was higher in the short-stay pathway, but patient-reported outcomes were similar for the two care groups [79]. The Enhanced Recovery After Surgery (ERAS®) Society recommendations for perioperative joint replacement care are consensus-based (rather than consistently evidence-based) [21] without patient selection specifications, likely due to a lack of high-quality evidence on this aspect.

### Strengths and limitations

Strengths of this systematic review include a comprehensive search of the literature across multiple evidence databases, standardised risk of bias appraisal, assessment of evidence certainty and pooled analysis of key safety indicators by study design (both during and after the hospital admission). The results are likely to be broadly generalisable as the included studies were conducted in both middle- and high-income countries and in a variety of healthcare settings including metropolitan and non-metropolitan hospitals, teaching hospitals and military-based hospitals.

In accordance with our review protocol, we did not plan to assess the cost of short-stay programmes, length of stay or adherence to short-stay components in relation to safety or patient outcomes as these aspects have been assessed in previous reviews [14, 80, 81]. We also only examined harms and so the differences between short-stay and usual care participants may have been overestimated where present. As infections and wound complications were inconsistently reported across the included studies, it was not feasible to further categorise these outcomes. We excluded single-group cohort studies but recognise that additional data may be available from this research. Articles published in languages other than English were also excluded from this review (four potentially relevant studies published in Chinese were excluded in the title and abstract screening and full-text review stages). Based on the similarities of published data in English, we do not anticipate that this would have altered our conclusions. We also note that the review included four studies from China that were published in English, giving representation to short-stay joint replacement research conducted in this country.

### Implications for clinical practice

This review has identified that there is insufficient high-quality trial evidence to support the 90-day safety profile of short-stay joint replacement programmes compared to usual care. Short-stay programmes may have non-inferior safety outcomes (for hospital readmission, blood loss, other complications, neurovascular injury, and stiffness outcomes) compared to usual care, but due to the small number of RCTs, small sample sizes and low event rates, the certainty of this evidence is low. There was no evidence of significant harms (with respect to reoperations, blood transfusion requirements, emergency department visits, infection, mortality, periprosthetic fractures, VTE, wound complications, or dislocation) in the quasi-experimental studies but due to lower levels of evidence we cannot be confident in these findings. Further evidence is required to determine whether short-stay programmes are safer than usual care pathways. This is time critical, given the increasing use of short-stay joint replacement programmes in many international jurisdictions, and the need for evidence-based decisions around resource allocation.

A cluster RCT including different hospital settings (for example, public and private hospitals) could be established to address this important yet unanswered research question. The trial could test a mandated length of stay (for example, 2–3 days) with standardised pre-operative, intra-operative, post-operative and post-discharge multidisciplinary protocols. Efficacy, safety and process outcomes could be evaluated, and the trial would also provide critical (and currently unavailable) data on patient and clinical factors that predict successful discharge home. Efforts to standardise the selection and reporting of safety and patient-related outcomes in short-stay joint replacement research would also facilitate future pooling and analysis of these data.

## Conclusions

There is low certainty evidence that short-stay programmes for THR and KR may have non-inferior 90-day safety outcomes, compared to usual care. Most of the included studies used quasi-experimental designs and further evidence from high-quality RCTs is needed to determine whether short-stay programmes are safer than usual care pathways. There remains an important evidence gap around factors associated with poor outcomes, to guide optimal patient selection into short-stay programmes.

### Supplementary Information

**Additional file 1.**

**Additional file 2.**

**Additional file 3.**

**Additional file 4.**

**Additional file 5.**

## Data Availability

All relevant data are reported in this paper and the supplementary files.

## Abbreviations

AOANJRR: Australian Orthopaedic Association National Joint Replacement Registry
CENTRAL: Cochrane Central Register of Controlled Trials
CI: Confidence interval
CINAHL: Cumulative Index to Nursing and Allied Health Literature
DVT: Deep vein thrombosis
HR: Hazard ratio
IQR: Interquartile range
JBI: Joanna Briggs Institute
KR: Knee replacement
OECD: Organisation for Economic Co-operation and Development
OR: Odds ratio
PE: Pulmonary embolism
PRISMA: Preferred Reporting Items for Systematic Reviews and Meta-analyses
PROM: Patient-reported outcome measure
RCT: Randomised controlled trial
SD: Standard deviation
THR: Total hip replacement
TKR: Total knee replacement
UK: United Kingdom
USD: US dollars
VTE: Venous thromboembolism

## References

[1] Kurtz S, Ong K, Lau E, Mowat F, Halpern M (2007). Projections of primary and revision hip and knee arthroplasty in the United States from 2005 to 2030. J Bone Joint Surg Am.

[2] Long H, Liu Q, Yin H, Wang K, Diao N, Zhang Y (2022). Prevalence trends of site-specific osteoarthritis from 1990 to 2019: Findings from the Global Burden of Disease Study 2019. Arthritis Rheumatol.

[3] Yahaya I, Wright T, Babatunde OO, Corp N, Helliwell T, Dikomitis L (2021). Prevalence of osteoarthritis in lower middle- and low-income countries: A systematic review and meta-analysis. Rheumatol Int.

[4] OECD iLibrary. Hip and knee replacement 2021. https://www.oecd-ilibrary.org/sites/8b492d7a-en/index.html?itemId=/content/component/8b492d7a-en. Accessed 13 April 2023.

[5] Australian Orthopaedic Association National Joint Replacement Registry (AOANJRR). Hip, Knee & Shoulder Arthroplasty: 2022 Annual Report. Adelaide: Australian Orthopaedic Association; 2022.

[6] National Joint Registry. 19th Annual Report 2022: Surgical data to 31 December 2021. United Kingdom; 2022.

[7] Hoffmann JD, Kusnezov NA, Dunn JC, Zarkadis NJ, Goodman GP, Berger RA (2018). The shift to same-day outpatient joint arthroplasty: A systematic review. J Arthroplasty.

[8] Morrell AT, Layon DR, Scott MJ, Kates SL, Golladay GJ, Patel NK (2021). Enhanced recovery after primary total hip and knee arthroplasty: A systematic review. J Bone Joint Surg Am.

[9] Qurashi S, Bajwa S, Aktas S, Bestic W, Chinnappa J (2021). Overnight or short stay joint replacements in the public and private settings: An Australian experience. Recon Rev.

[10] Vendittoli PA, Pellei K, Desmeules F, Masse V, Loubert C, Lavigne M (2019). Enhanced recovery short-stay hip and knee joint replacement program improves patients outcomes while reducing hospital costs. Orthop Traumatol Surg Res.

[11] Petersen PB, Kehlet H, Jorgensen CC, Lundbeck Foundation Centre for Fast-track H, Knee Replacement Collaborative G (2020). Improvement in fast-track hip and knee arthroplasty: A prospective multicentre study of 36,935 procedures from 2010 to 2017. Sci Rep.

[12] Schultz BJ, Segovia N, Castillo TN (2019). Successful implementation of an accelerated recovery and outpatient total joint arthroplasty program at a county hospital. J Am Acad Orthop Surg Glob Res Rev.

[13] Fatima M, Scholes CJ, Tutty A, Ebrahimi M, Genon M, Martin SJ (2022). Patient-reported outcomes of a short hospital stay after total knee replacement in a regional public hospital: a prospective cohort treated 2018–2019. ANZ J Surg.

[14] Pritchard MG, Murphy J, Cheng L, Janarthanan R, Judge A, Leal J (2020). Enhanced recovery following hip and knee arthroplasty: A systematic review of cost-effectiveness evidence. BMJ Open.

[15] Zhu S, Qian W, Jiang C, Ye C, Chen X (2017). Enhanced recovery after surgery for hip and knee arthroplasty: A systematic review and meta-analysis. Postgrad Med J.

[16] Page MJ, Moher D, Bossuyt PM, Boutron I, Hoffmann TC, Mulrow CD (2021). PRISMA 2020 explanation and elaboration: Updated guidance and exemplars for reporting systematic reviews. BMJ.

[17] U.S. National Library of Medicine. ClinicalTrials.gov: NIH; 2023 https://clinicaltrials.gov. Accessed 29 May 2023.

[18] Joanna Briggs Institute. Critical Appraisal Tools Adelaide: The University of Adelaide; 2023 https://jbi.global/critical-appraisal-tools. Accessed 13 April 2023.

[19] Barker TH, Stone JC, Sears K, Klugar M, Tufanaru C, Leonardi-Bee J (2023). The revised JBI critical appraisal tool for the assessment of risk of bias for randomized controlled trials. JBI Evid Synth.

[20] Sterne JAC, Hernan MA, McAlennan A, Reeves BC, Higgins JPT. Chapter 25: Assessing risk of bias in a non-randomized study 2022. In: Cochrane Handbook for Systematic Reviews of Interventions version 63 (updated February 2022). www.training.cochrane.org/handbook. Accessed 29 May 2023.

[21] Wainwright TW, Gill M, McDonald DA, Middleton RG, Reed M, Sahota O (2020). Consensus statement for perioperative care in total hip replacement and total knee replacement surgery: Enhanced Recovery After Surgery (ERAS®) Society recommendations. Acta Orthop.

[22] Deeks JJ, Higgins JPT, Altman DGe. Chapter 10: Analysing data and undertaking meta-analyses. 2022. In: Cochrane Handbook for Systematic Reviews of Interventions version 63 (updated February 2022). https://training.cochrane.org/handbook/current/chapter-10. Accessed 13 April 2023.

[23] Schünemann HJ, Higgins JPT, Vist GE, Glasziou P, Akl EA, Skoetz N, et al. Chapter 14: Completing ‘Summary of findings’ tables and grading the certainty of the evidence. 2022. In: Cochrane Handbook for Systematic Reviews of Interventions version 63 (updated Feburary 2022). https://training.cochrane.org/handbook/current/chapter-14. Accessed 29 May 2023.

[24] GradePro.org. Handbook for grading the quality of evidence and the strength of recommendations using the GRADE approach. 2013. In: GRADE Handbook. GRADEPro. https://gdt.gradepro.org/app/handbook/handbook.html#h.trgki08omk7z. Accessed 29 May 2023.

[25] Arshad H, Royan S, Smith T, Barker L, Chirodian N, Wimhurst J (2014). Norwich Enhanced Recovery Programme vs. non-enhanced recovery following hip and knee replacement: A matched-cohort study. Int J Orthop Trauma Nurs.

[26] Dhawan R, Rajgor H, Yarlagadda R, John J, Graham NM (2017). Enhanced recovery protocol and hidden blood loss in patients undergoing total knee arthroplasty. Indian J Orthop.

[27] Dwyer AJ, Thomas W, Humphry S, Porter P (2014). Enhanced recovery programme for total knee replacement to reduce the length of hospital stay. J Orthop Surg.

[28] Khan SK, Malviya A, Muller SD, Carluke I, Partington PF, Emmerson KP (2014). Reduced short-term complications and mortality following Enhanced Recovery primary hip and knee arthroplasty: Results from 6,000 consecutive procedures. Acta Orthop.

[29] Maempel JF, Clement ND, Ballantyne JA, Dunstan E (2016). Enhanced recovery programmes after total hip arthroplasty can result in reduced length of hospital stay without compromising functional outcome. Bone Joint J.

[30] Maempel JF, Walmsley PJ (2015). Enhanced recovery programmes can reduce length of stay after total knee replacement without sacrificing functional outcome at one year. Ann R Coll Surg Engl.

[31] Malviya A, Martin K, Harper I, Muller SD, Emmerson KP, Partington PF (2011). Enhanced recovery program for hip and knee replacement reduces death rate. Acta Orthop.

[32] McDonald DA, Siegmeth R, Deakin AH, Kinninmonth AW, Scott NB (2012). An enhanced recovery programme for primary total knee arthroplasty in the United Kingdom - follow up at one year. Knee.

[33] Reilly KA, Beard DJ, Barker KL, Dodd CA, Price AJ, Murray DW (2005). Efficacy of an accelerated recovery protocol for Oxford unicompartmental knee arthroplasty - a randomised controlled trial. Knee.

[34] Savaridas T, Serrano-Pedraza I, Khan SK, Martin K, Malviya A, Reed MR (2013). Reduced medium-term mortality following primary total hip and knee arthroplasty with an enhanced recovery program. A study of 4,500 consecutive procedures. Acta Orthop.

[35] Starks I, Wainwright TW, Lewis J, Lloyd J, Middleton RG (2014). Older patients have the most to gain from orthopaedic enhanced recovery programmes. Age Ageing.

[36] Scott NB, McDonald D, Campbell J, Smith RD, Carey AK, Johnston IG (2013). The use of enhanced recovery after surgery (ERAS) principles in Scottish orthopaedic units–an implementation and follow-up at 1 year, 2010–2011: A report from the Musculoskeletal Audit. Scotland Arch Orthop Trauma Surg.

[37] Dwyer AJ, Tarassoli P, Thomas W, Porter P (2012). Enhanced recovery program in total hip arthroplasty. Indian J Orthop.

[38] Alvis BD, Amsler RG, Leisy PJ, Feng X, Shotwell MS, Pandharipande PP (2021). Effects of an anesthesia perioperative surgical home for total knee and hip arthroplasty at a Veterans Affairs Hospital: A quality improvement before-and-after cohort study. Can J Anaesth.

[39] Doman DM, Gerlinger TL (2012). Total joint arthroplasty cost savings with a rapid recovery protocol in a military medical center. Mil Med.

[40] Featherall J, Brigati DP, Faour M, Messner W, Higuera CA (2018). Implementation of a total hip arthroplasty care pathway at a high-volume health system: Effect on length of stay, discharge disposition, and 90-day complications. J Arthroplasty.

[41] Stambough JB, Nunley RM, Curry MC, Steger-May K, Clohisy JC (2015). Rapid recovery protocols for primary total hip arthroplasty can safely reduce length of stay without increasing readmissions. J Arthroplasty.

[42] Taylor AJ, Kay RD, Bryman JA, Tye EY, Longjohn DB, Najibi S (2022). Outcomes of an institutional rapid recovery protocol for total joint arthroplasty at a safety net hospital. J Am Acad Orthop Surg Glob Res Rev.

[43] Teeny SM, York SC, Benson C, Perdue ST (2005). Does shortened length of hospital stay affect total knee arthroplasty rehabilitation outcomes?. J Arthroplasty.

[44] Yanik JM, Bedard NA, Hanley JM, Otero JE, Callaghan JJ, Marsh JL (2018). Rapid recovery total joint arthroplasty is safe, efficient, and cost-effective in the Veterans Administration setting. J Arthroplasty.

[45] Chung MMT, Ng JKF, Ng FY, Chan PK, Chiu KY (2021). Effects of enhanced recovery after surgery practices on postoperative recovery and length of stay after unilateral primary total hip or knee arthroplasty in a private hospital. Hong Kong Med J.

[46] Jiang HH, Jian XF, Shangguan YF, Qing J, Chen LB (2019). Effects of enhanced recovery after surgery in total knee arthroplasty for patients older than 65 years. Orthop Surg.

[47] Liao X, Xu X (2022). The effect of cold therapy combined with ERAS in the postoperative care of patients undergoing total knee arthroplasty. Am J Transl Res.

[48] Wang X, Chen Y, Zhao J, Wang B, Chen Z (2023). Enhanced recovery after surgery for primary total hip arthroplasty: Analysis of post-operative blood indexes. Int Orthop.

[49] Harkouk H, Capmas P, Derridj N, Belbachir A, Nkam L, Aegerter P (2021). Limited impact of a top-down approach to improve enhanced recovery programme in French university hospitals: A before-after retrospective survey. Perioper Med.

[50] Picart B, Lecoeur B, Rochcongar G, Dunet J, Pegoix M, Hulet C (2021). Implementation and results of an enhanced recovery (fast-track) program in total knee replacement patients at a French university hospital. Orthop Traumatol Surg Res.

[51] Slim K, Boudemaghe T, Delaunay L, Leger L, Bizard F (2022). Favorable effect of enhanced recovery programs on post-discharge mortality: A French nationwide study. Perioper Med.

[52] Castorina S, Guglielmino C, Castrogiovanni P, Szychlinska MA, Ioppolo F, Massimo P (2017). Clinical evidence of traditional vs fast track recovery methodologies after total arthroplasty for osteoarthritis knee treatment. A retrsopective observational study MLTJ.

[53] Romano LU, Rigoni M, Torri E, Nella M, Morandi M, Casetti P (2021). A propensity score-matched analysis to assess the outcomes in pre- and post-fast-track hip and knee elective prosthesis patients. J Clin Med.

[54] Tasso F, Simili V, Di Matteo B, Monteleone G, Martorelli F, De Angelis A (2022). A rapid recovery protocol for hip and knee replacement surgery: A report of the outcomes in a referral center. Eur Rev Med Pharmacol Sci.

[55] den Hartog YM, Mathijssen NM, Vehmeijer SB (2013). Reduced length of hospital stay after the introduction of a rapid recovery protocol for primary THA procedures. Acta Orthop.

[56] Didden AGM, Punt IM, Feczko PZ, Lenssen AF (2019). Enhanced recovery in usual health care improves functional recovery after total knee arthroplasty. Int J Orthop Trauma Nurs.

[57] Fransen BL, Hoozemans MJM, Argelo KDS, Keijser LCM, Burger BJ (2018). Fast-track total knee arthroplasty improved clinical and functional outcome in the first 7 days after surgery: A randomized controlled pilot study with 5-year follow-up. Arch Orthop Trauma Surg.

[58] Gleicher Y, Siddiqui N, Mazda Y, Matelski J, Backstein DJ, Wolfstadt JI (2021). Reducing acute hospitalization length of stay after total knee arthroplasty: A quality improvement study. J Arthroplasty.

[59] Raphael M, Jaeger M, van Vlymen J (2011). Easily adoptable total joint arthroplasty program allows discharge home in two days. Can J Anaesth.

[60] Larsen K, Hvass KE, Hansen TB, Thomsen PB, Soballe K (2008). Effectiveness of accelerated perioperative care and rehabilitation intervention compared to current intervention after hip and knee arthroplasty. A before-after trial of 247 patients with a 3-month follow-up. BMC Musculoskelet Disord.

[61] Petersen MK, Madsen C, Andersen NT, Soballe K (2006). Efficacy of multimodal optimization of mobilization and nutrition in patients undergoing hip replacement: A randomized clinical trial. Acta Anaesthesiol Scand.

[62] Gwynne-Jones DP, Martin G, Crane C (2017). Enhanced recovery after surgery for hip and knee replacements. Orthop Nurs.

[63] Stowers MD, Manuopangai L, Hill AG, Gray JR, Coleman B, Munro JT (2016). Enhanced Recovery After Surgery in elective hip and knee arthroplasty reduces length of hospital stay. ANZ J Surg.

[64] Berg U, A WD, Nilsdotter A, Naucler E, Sundberg M, Rolfson O (2021). Fast-track programs in total hip and knee replacement at Swedish hospitals - Influence on 2-year risk of revision and mortality. J Clin Med.

[65] Berg U, BuLow E, Sundberg M, Rolfson O (2018). No increase in readmissions or adverse events after implementation of fast-track program in total hip and knee replacement at 8 Swedish hospitals: An observational before-and-after study of 14,148 total joint replacements 2011–2015. Acta Orthop.

[66] Joo B, Marquez J, Model G, Fan B, Osmotherly PG (2022). Impact of a new post-operative care model in a rural hospital after total hip replacement and total knee replacement. Aust J Rural Health.

[67] de Carvalho Almeida RF, Serra HO, de Oliveira LP (2021). Fast-track versus conventional surgery in relation to time of hospital discharge following total hip arthroplasty: A single-center prospective study. J Orthop Surg Res.

[68] Reinhard J, Schindler M, Leiss F, Greimel F, Grifka J, Benditz A (2023). No clinically significant difference in postoperative pain and side effects comparing conventional and enhanced recovery total hip arthroplasty with early mobilization. Arch Orthop Trauma Surg.

[69] Azam MQ, Goyal T, Paul S, Yadav AK, Govil N (2022). Enhanced recovery protocol after single-stage bilateral primary total knee arthroplasty decreases duration of hospital stay without increasing complication rates. Eur J Orthop Surg Traumatol.

[70] Galbraith JG, Fenelon C, Gibbons J, Kelly GA, Bennett D (2017). Enhanced recovery in lower limb arthroplasty in the Irish setting. Ir J Med Sci.

[71] Amlie E, Lerdal A, Gay CL, Hovik O, Nordsletten L, Dimmen S (2016). A trend for increased risk of revision surgery due to deep infection following fast-track hip arthroplasty. Adv Orthop.

[72] Ripolles-Melchor J, Abad-Motos A, Diez-Remesal Y, Aseguinolaza-Pagola M, Padin-Barreiro L, Sanchez-Martin R (2020). Association between use of Enhanced Recovery After Surgery Protocol and postoperative complications in total hip and knee arthroplasty in the postoperative outcomes within Enhanced Recovery after Surgery Protocol in Elective Total Hip and Knee Arthroplasty Study (POWER2). JAMA Surg.

[73] Edelmann L, Hempel M, Podsiadlo N, Schweizer N, Tong C, Galvain T (2022). Reduced length of stay following patient pathway optimization for primary hip and knee arthroplasty at a Swiss Hospital. Clin Econ Outcomes Res.

[74] McCann-Spry L, Pelton J, Grandy G, Newell D (2016). An interdisciplinary approach to reducing length of stay in joint replacement patients. Orthop Nurs.

[75] Hill AM, Ross-Adjie G, McPhail SM, Jacques MA, Bulsara M, Cranfield A (2022). Incidence and associated risk factors for falls in older adults after elective total knee replacement surgery: A prospective cohort study. Am J Phys Med Rehabil.

[76] Liu Y, Yang Y, Liu H, Wu W, Wu X, Wang T (2020). A systematic review and meta-analysis of fall incidence and risk factors in elderly patients after total joint arthroplasty. Medicine.

[77] James SL, Lucchesi LR, Bisignano C, Castle CD, Dingels ZV, Fox JT (2020). The global burden of falls: Global, regional and national estimates of morbidity and mortality from the Global Burden of Disease Study 2017. Inj Prev.

[78] Jones EL, Wainwright TW, Foster JD, Smith JR, Middleton RG, Francis NK (2014). A systematic review of patient reported outcomes and patient experience in enhanced recovery after orthopaedic surgery. Ann R Coll Surg Engl.

[79] Hardy A, Courgeon M, Pellei K, Desmeules F, Loubert C, Vendittoli PA (2022). Improved clinical outcomes of outpatient enhanced recovery hip and knee replacements in comparison to standard inpatient procedures: A study of patients who experienced both. Orthop Traumatol Surg Res.

[80] Christelis N, Wallace S, Sage CE, Babitu U, Liew S, Dugal J (2015). An enhanced recovery after surgery program for hip and knee arthroplasty. Med J Aust.

[81] Liu SY, Li C, Zhang PX (2021). Enhanced recovery after surgery for hip fractures: A systematic review and meta-analysis. Perioper Med.

