# Supplementary material for: A systematic review and meta-analysis of short-stay programmes for total hip and knee replacement, focusing on safety and optimal patient selection
Source: BMC Med. 2023 Dec 21;21:511. doi: 10.1186/s12916-023-03219-5 (PMC10740291; doi:10.1186/s12916-023-03219-5)
Supplement: Supplementary file 2 — Additional file 2. [file 12916_2023_3219_MOESM2_ESM.docx]

**Supplementary File 2. Search Strategies and Yields**

**Search 1: 2000 - August 2022**

|  | **Ovid MEDLINE** | **CINAHL** | **Cochrane** | **EMBASE** |
| --- | --- | --- | --- | --- |
| 1 | exp Arthroplasty, Replacement, Hip/ | (MH "Arthroplasty, Replacement, Hip") | exp Arthroplasty, Replacement, Hip/ | exp hip arthroplasty/ or exp total hip replacement/ or hip prosthesis/ or exp total hip prosthesis/ or exp hip replacement/ |
| 2 | exp Arthroplasty, Replacement, Knee/ | (MH "Arthroplasty, Replacement, Knee") OR (MH "Arthroplasty, Knee, Unicompartmental") | exp Arthroplasty, Replacement, Knee/ | exp total knee prosthesis/ or exp knee prosthesis/ or exp knee arthroplasty/ or exp total knee arthroplasty/ or exp knee replacement/ |
| 3 | (total hip replacement or hip replacement or hip arthroplasty or hip prosthesis).mp. | TI (("total hip replacement" OR "hip replacement" OR "hip arthroplasty" OR "hip prosthesis")) OR AB (("total hip replacement" OR "hip replacement" OR "hip arthroplasty" OR "hip prosthesis")) | (total hip replacement or hip replacement or hip arthroplasty or hip prosthesis).mp. | (total hip replacement or hip replacement or hip arthroplasty or hip prosthesis).mp. |
| 4 | (total knee replacement or knee replacement or knee arthroplasty or knee prosthesis).mp. | TI ((total knee replacement OR knee replacement OR knee arthroplasty OR knee prosthesis)) OR AB ((total knee replacement OR knee replacement OR knee arthroplasty OR knee prosthesis)) | (total knee replacement or knee replacement or knee arthroplasty or knee prosthesis).mp. | (total knee replacement or knee replacement or knee arthroplasty or knee prosthesis).mp. |
| 5 | 1 or 2 or 3 or 4 | S1 OR S2 OR S3 OR S4 | 1 or 2 or 3 or 4 | 1 or 2 or 3 or 4 |
| 6 | exp Enhanced Recovery After Surgery/ | (MH "Enhanced Recovery After Surgery") | exp Enhanced Recovery After Surgery/ | exp enhanced recovery after surgery/ |
| 7 | (enhanced recovery* or ERAS or short stay or fast track or FTS or accelerated discharge or early discharge or rapid recovery).mp. | (MH "Early Patient Discharge") | (enhanced recovery* or ERAS or short stay or fast track or FTS or accelerated discharge or early discharge or rapid recovery).mp. | exp hospital discharge/ |
| 8 | 6 or 7 | TI ((enhanced recovery* OR ERAS OR short stay OR fast track OR FTS OR accelerated discharge OR early discharge OR rapid recovery)) OR AB ((enhanced recovery* OR ERAS OR short stay OR fast track OR FTS OR accelerated discharge OR early discharge OR rapid recovery)) | 6 or 7 | (enhanced recovery* or ERAS or short stay or fast track or FTS or accelerated discharge or early discharge or rapid recovery).mp. |
| 9 | 5 and 8 | S6 OR S7 OR S8 | 5 and 8 | 6 or 7 or 8 |
| 10 |  | S5 AND S9 |  | 5 and 9 |
| **Yield** | **809** | **726** | **351** | **3,525** |

**Search 2: 2022 – May 2023**

|  | **Ovid MEDLINE** | **CINAHL** | **Cochrane** | **EMBASE** |
| --- | --- | --- | --- | --- |
| 1 | exp Arthroplasty, Replacement, Hip/ | (MH "Arthroplasty, Replacement, Hip") | exp Arthroplasty, Replacement, Hip/ | exp hip arthroplasty/ or exp total hip replacement/ or hip prosthesis/ or exp total hip prosthesis/ or exp hip replacement/ |
| 2 | exp Arthroplasty, Replacement, Knee/ | (MH "Arthroplasty, Replacement, Knee") OR (MH "Arthroplasty, Knee, Unicompartmental") | exp Arthroplasty, Replacement, Knee/ | exp total knee prosthesis/ or exp knee prosthesis/ or exp knee arthroplasty/ or exp total knee arthroplasty/ or exp knee replacement/ |
| 3 | (total hip replacement or hip replacement or hip arthroplasty or hip prosthesis).mp. | TI (("total hip replacement" OR "hip replacement" OR "hip arthroplasty" OR "hip prosthesis")) OR AB (("total hip replacement" OR "hip replacement" OR "hip arthroplasty" OR "hip prosthesis")) | (total hip replacement or hip replacement or hip arthroplasty or hip prosthesis).mp. | (total hip replacement or hip replacement or hip arthroplasty or hip prosthesis).mp. |
| 4 | (total knee replacement or knee replacement or knee arthroplasty or knee prosthesis).mp. | TI ((total knee replacement OR knee replacement OR knee arthroplasty OR knee prosthesis)) OR AB ((total knee replacement OR knee replacement OR knee arthroplasty OR knee prosthesis)) | (total knee replacement or knee replacement or knee arthroplasty or knee prosthesis).mp. | (total knee replacement or knee replacement or knee arthroplasty or knee prosthesis).mp. |
| 5 | 1 or 2 or 3 or 4 | S1 OR S2 OR S3 OR S4 | 1 or 2 or 3 or 4 | 1 or 2 or 3 or 4 |
| 6 | exp Enhanced Recovery After Surgery/ | (MH "Enhanced Recovery After Surgery") | exp Enhanced Recovery After Surgery/ | exp enhanced recovery after surgery/ |
| 7 | (enhanced recovery* or ERAS or short stay or fast track or FTS or accelerated discharge or early discharge or rapid recovery).mp. | (MH "Early Patient Discharge") | (enhanced recovery* or ERAS or short stay or fast track or FTS or accelerated discharge or early discharge or rapid recovery).mp. | exp hospital discharge/ |
| 8 | 6 or 7 | TI ((enhanced recovery* OR ERAS OR short stay OR fast track OR FTS OR accelerated discharge OR early discharge OR rapid recovery)) OR AB ((enhanced recovery* OR ERAS OR short stay OR fast track OR FTS OR accelerated discharge OR early discharge OR rapid recovery)) | 6 or 7 | (enhanced recovery* or ERAS or short stay or fast track or FTS or accelerated discharge or early discharge or rapid recovery).mp. |
| 9 | 5 and 8 | S6 OR S7 OR S8 | 5 and 8 | 6 or 7 or 8 |
| 10 |  | S5 AND S9 |  | 5 and 9 |
| **Yield** | **103** | **71** | **41** | **561** |
