# Supplementary material for: A systematic review and meta-analysis of short-stay programmes for total hip and knee replacement, focusing on safety and optimal patient selection
Source: BMC Med. 2023 Dec 21;21:511. doi: 10.1186/s12916-023-03219-5 (PMC10740291; doi:10.1186/s12916-023-03219-5)
Supplement: Supplementary file 3 — Additional file 3. [file 12916_2023_3219_MOESM3_ESM.docx]

| **Study** | **Selection and Allocation** | | | **Administration of Intervention / Exposure** | | | **Assessment, Detection, and Measurement of the Outcome** | | | **Participant Retention** | **Statistical Conclusion Validity** | | |
| --- | --- | --- | --- | --- | --- | --- | --- | --- | --- | --- | --- | --- | --- |
|  | 1. Was true randomisation used for assignment of participants to treatment groups? | 2. Was allocation to treatment groups concealed? | 3. Were treatment groups similar at baseline? | 4. Were participants blind to treatment assignment? | 5. Were those delivering the intervention blind to treatment assignment? | 6. Were outcome assessors blind to treatment assignment? | 7. Were treatment groups treated identically other than the intervention of interests? | 8. Was follow-up complete and if not, were differences between groups in terms of their follow up adequately described and analyzed? | 9. Were participants analysed in the groups to which they were randomised? | 10. Were outcomes measured in the same way for treatment groups? | 11. Were outcomes measured in a reliable way? | 12. Was appropriate statistical analysis used? | 13. Was the trial design appropriate, and any deviations from the standard RCT design accounted for in the conduct and analysis of the trial? |
| Fransen  2018 | Yes | No | Yes | No | No | No | Yes | Unclear | Yes | Yes | Unclear | Yes | Yes |
| Petersen  2006 | Yes | No | Yes | No | No | No | Yes | Yes | Yes | Yes | Unclear | Yes | Yes |
| Reilly  2005 | Yes | No | Yes | Unclear | No | Unclear | Yes | Yes | Yes | Yes | Yes | Yes | Yes |

**Supplementary File 3. Risk of Bias**

**Joanna Briggs Institute Risk of Bias Tool for Randomised Controlled Trials**

**Joanna Briggs Institute Risk of Bias Tool for Quasi-Experimental Studies**

| **Study** | **Temporal Relationship of the Variables** | **Selection Bias** | | **Control Group** | **Multiple Measurements of the Outcome** | **Loss to Follow-Up** | **Statistical Conclusion Validity** | | |
| --- | --- | --- | --- | --- | --- | --- | --- | --- | --- |
|  | 1.Is it clear in the study what is the ‘cause’ and what is the ‘effect’ (i.e. there is no confusion about which variable comes first)? | 2. Were the participants included in any comparisons similar? | 3. Were the participants included in any comparisons receiving similar treatment/care, other than the exposure or intervention of interest? | 4. Was there a control group? | 5. Were there multiple measurements of the outcome both pre and post the intervention/exposure? | 6.Was follow up complete and if not, were differences between groups in terms of their follow up adequately described and analyzed? | 7. Were the outcomes of participants included in any comparisons measured in the same way? | 8. Were outcomes measured in a reliable way? | 9. Was appropriate statistical analysis used? |
| Alvis 2021 | Yes | Yes | Yes | Yes | Not applicable | Yes | Yes | Yes | Yes |
| Amlie 2016 | Yes | Unclear | Yes | Yes | Not applicable | Unclear | Yes | Yes | Yes |
| Arshad 2014 | Yes | No | Yes | Yes | Not applicable | Yes | Yes | Yes | Yes |
| Azam 2022 | Yes | Yes | Yes | Yes | Not applicable | Yes | Yes | Unclear | Yes |
| Berg 2018 | Yes | No | Yes | Yes | Yes | Unclear | Yes | Yes | Yes |
| Berg 2021 | Yes | No | Yes | Yes | Not applicable | No | Yes | Yes | Yes |
| Castorina 2017 | Yes | No | Unclear | Yes | Unclear | Unclear | Yes | Yes | No |
| Chung 2021 | Yes | Yes | Yes | Yes | Not applicable | Yes | Unclear | Unclear | Yes |
| De Carvalho Almeida 2021 | Yes | Yes | Yes | Yes | Not applicable | Yes | Yes | Unclear | Yes |
| Den Hartog 2013 | Yes | Yes | No | Yes | Not applicable | Yes | Yes | Unclear | Yes |
| Dhawan 2017 | Yes | No | Yes | Yes | Not applicable | Unclear | Yes | Yes | Yes |
| Didden 2019 | Yes | Yes | Yes | Yes | No | Unclear | Yes | Yes | Yes |
| Doman 2012 | Yes | No | Yes | Yes | No | Yes | Yes | Yes | Yes |
| Dwyer 2012 | Yes | No | Yes | Yes | Not applicable | Unclear | Unclear | Unclear | Unclear |
| Dwyer 2014 | Yes | Yes | Yes | Yes | Not applicable | Yes | Yes | Yes | Yes |
| Edelmann 2022 | Yes | No | Yes | Yes | Not applicable | Yes | Yes | Yes | Yes |
| Featherall 2018 | Yes | No | Yes | Yes | Not applicable | Yes | Yes | Yes | Yes |
| Galbraith 2017 | Yes | Yes | Yes | Yes | Not applicable | Unclear | Yes | Yes | Unclear |
| Gleicher 2021 | Yes | Yes | Yes | Yes | Not applicable | Yes | Yes | Yes | Unclear |
| Gwynne-Jones 2017 | Yes | Yes | Yes | Yes | No | Yes | Yes | Yes | Unclear |
| Harkouk 2021 | Yes | No | Unclear | Yes | Not applicable | Yes | Yes | Yes | Yes |
| Jiang 2019 | Yes | Yes | Yes | Yes | Yes | No | Yes | Yes | No |
| Joo 2022 | Yes | Yes | Yes | Yes | Not applicable | No | Yes | Unclear | Yes |
| Khan 2014 | Yes | No | Yes | Yes | Not applicable | No | Yes | Yes | Yes |
| Liao 2022 | Yes | Yes | Yes | Yes | Yes | Yes | Yes | Yes | Yes |
| Larsen 2008 | Yes | Yes | Yes | Yes | Not applicable | Yes | Yes | Yes | Yes |
| Maempel 2015 | Yes | Yes | Yes | Yes | Yes | Yes | Yes | Unclear | Yes |
| Maempel 2016 | Yes | No | Yes | Yes | Yes | No | Yes | Yes | Yes |
| Malviya 2011 | Yes | No | Yes | Yes | Yes | Unclear | Yes | Yes | Yes |
| McDonald 2012 | Yes | Yes | Yes | Yes | Yes | Yes | Yes | Yes | Yes |
| Picart 2021 | Yes | Yes | Yes | Yes | Not applicable | Yes | Yes | Yes | Yes |
| Raphael 2011 | Yes | No | Yes | Yes | No | No | Yes | Yes | Yes |
| Ripolles-Melchor 2020 | Yes | No | Unclear | Yes | Not applicable | Unclear | Yes | Yes | Yes |
| Reinhard 2023 | Yes | Yes | Yes | Yes | Not applicable | Unclear | Unclear | Unclear | Yes |
| Romano 2021 | Yes | Yes | Yes | Yes | Not applicable | Yes | Yes | Yes | Yes |
| Savaridas 2013 | Yes | No | Yes | Yes | Not applicable | Yes | Yes | Yes | Yes |
| Scott 2013 | Yes | No | Yes | Yes | Not applicable | No | Unclear | Unclear | Unclear |
| Slim 2022 | Unclear | Yes | Unclear | Yes | Not applicable | Yes | Yes | Unclear | Yes |
| Stambough 2015 | Yes | No | Yes | Yes | Not applicable | Yes | Yes | Yes | Yes |
| Starks 2014 | Yes | Unclear | Yes | Yes | Not applicable | Yes | Yes | Unclear | No |
| Stowers 2016 | Yes | Yes | Yes | Yes | Not applicable | Unclear | Yes | Yes | No |
| Tasso 2022 | Yes | Yes | Yes | Yes | Yes | Unclear | Yes | Yes | No |
| Taylor 2022 | Yes | Yes | Yes | Yes | Not applicable | Yes | Yes | Yes | Yes |
| Teeny 2005 | Yes | Yes | Yes | Yes | Yes | No | Yes | Yes | Yes |
| Wang 2022 | Yes | No | Yes | Yes | Yes | Unclear | Yes | Yes | No |
| Yanik 2018 | Yes | No | Yes | Yes | Not applicable | Yes | Yes | Unclear | Yes |
