# Supplementary material for: A systematic review and meta-analysis of short-stay programmes for total hip and knee replacement, focusing on safety and optimal patient selection
Source: BMC Med. 2023 Dec 21;21:511. doi: 10.1186/s12916-023-03219-5 (PMC10740291; doi:10.1186/s12916-023-03219-5)
Supplement: Supplementary file 4 — Additional file 4. [file 12916_2023_3219_MOESM4_ESM.docx]

**Supplementary File 4. Meta-Analysis Forest Plots**

**
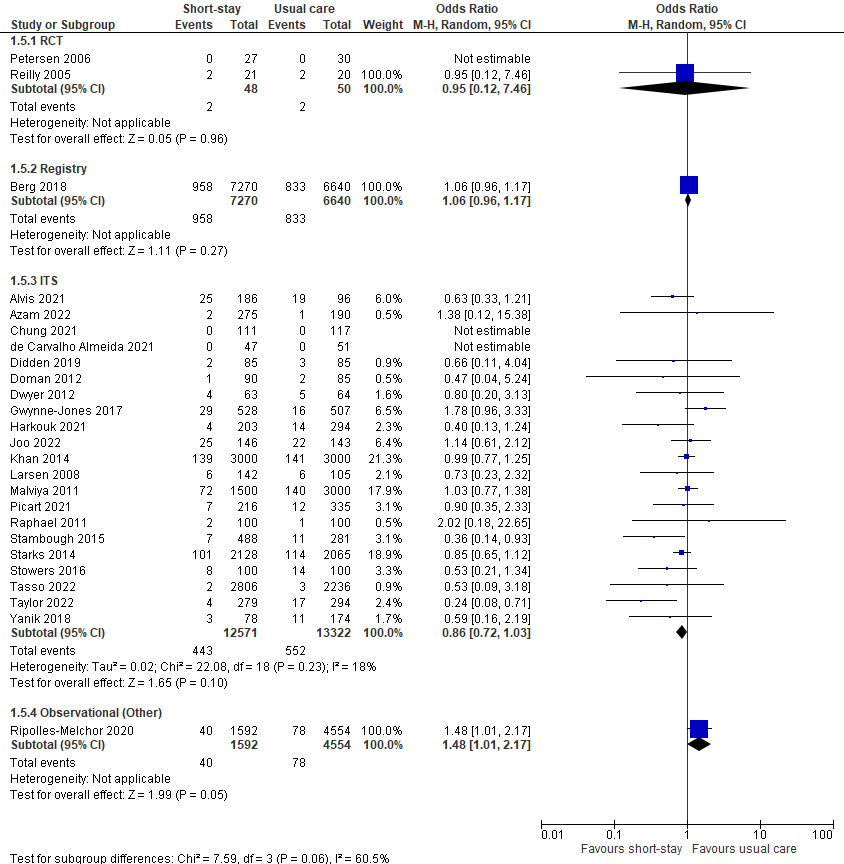
Readmissions**

**
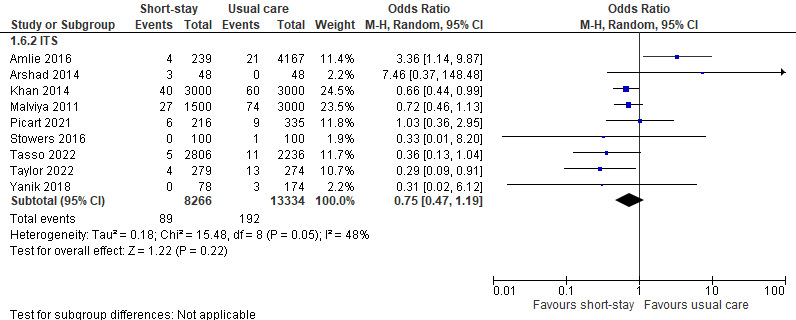
Reoperations**

**
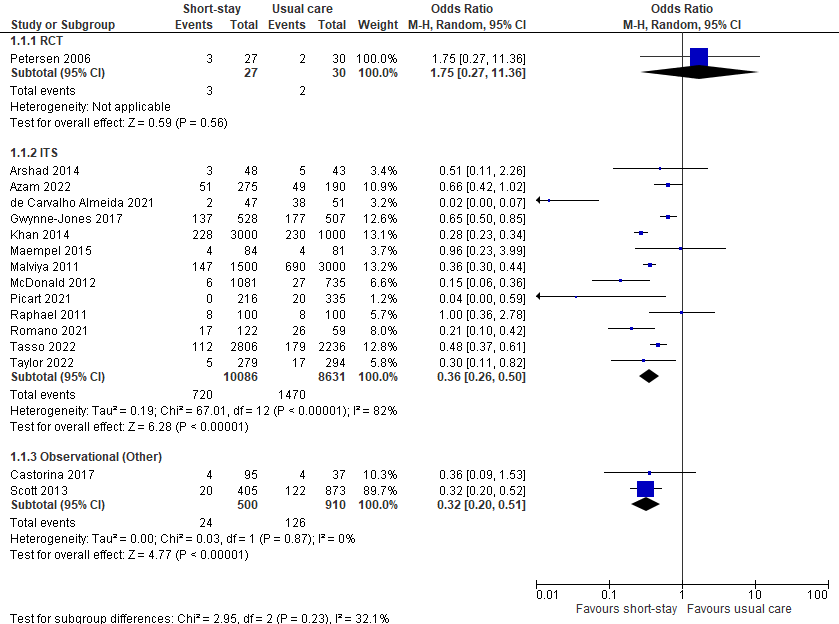
Requires Blood Transfusion**

**
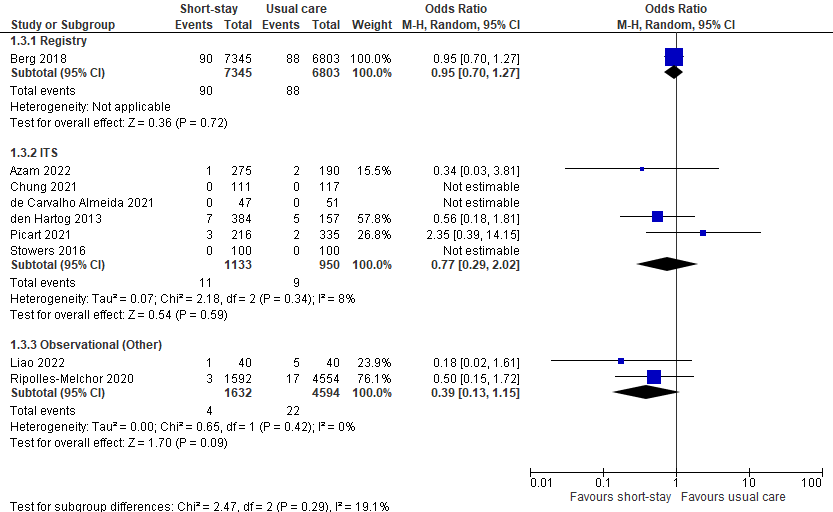
Infection**

**
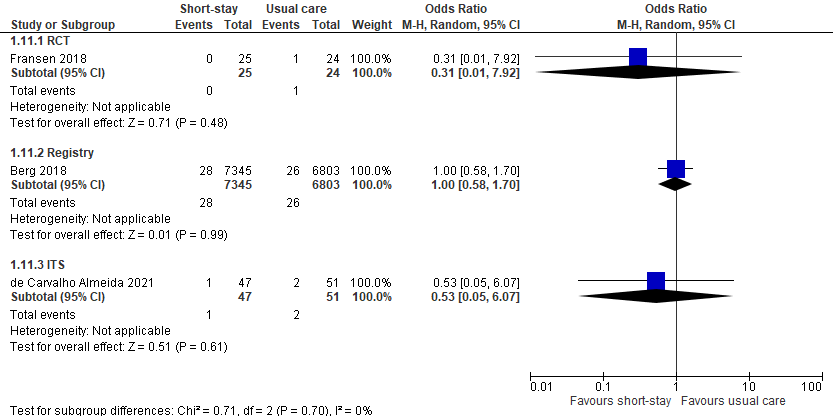
Neurovascular Injury**

**
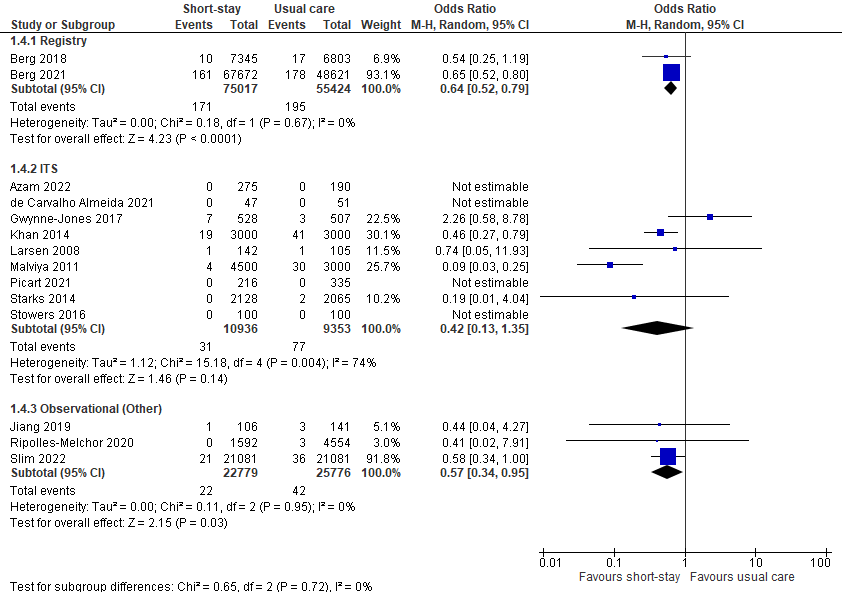
Mortality**

**
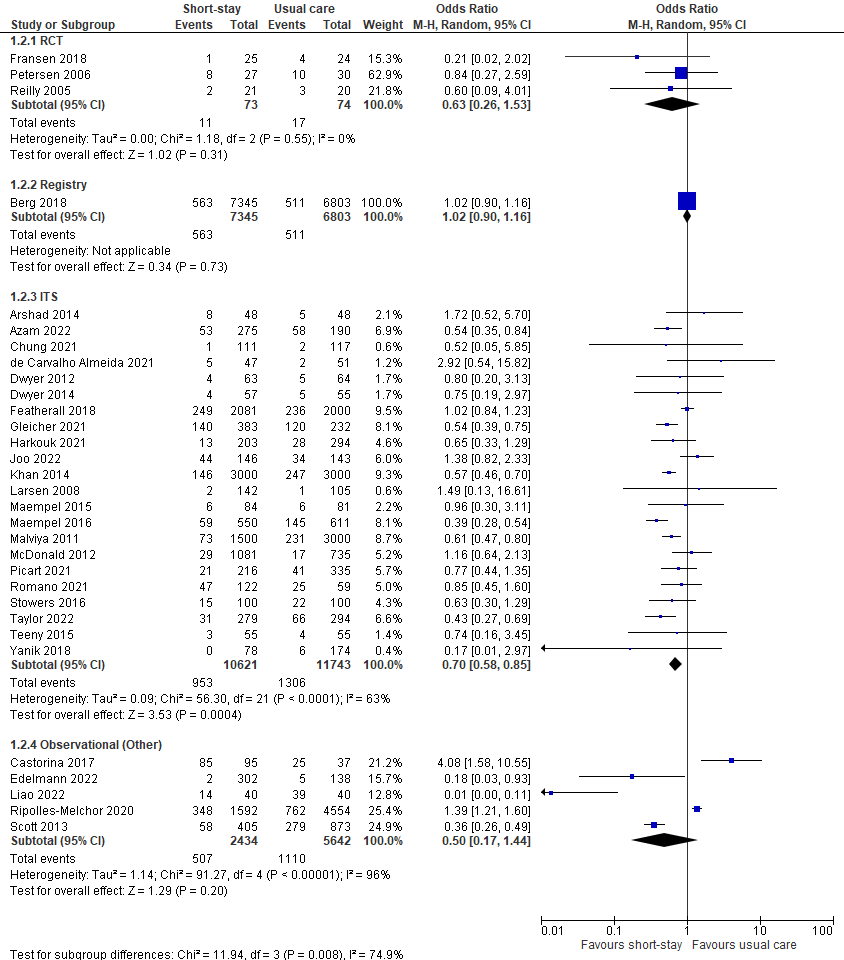
Other Complications**

**
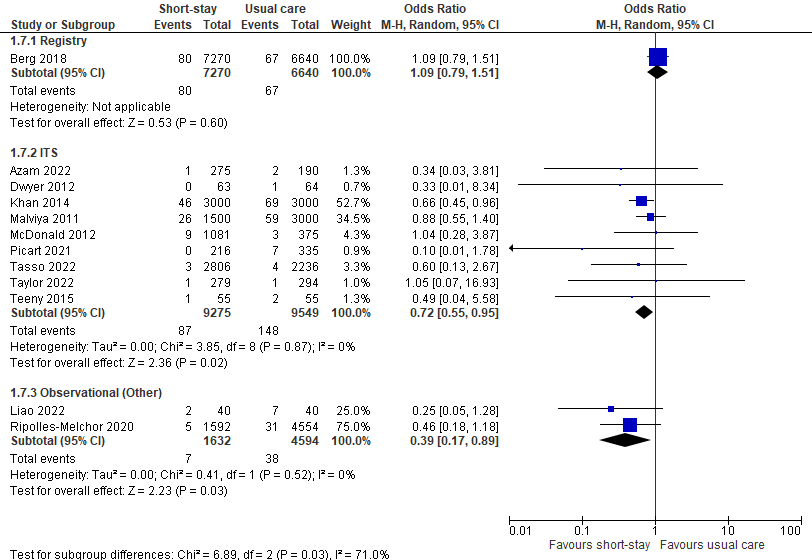
Venous Thromboembolism**

**
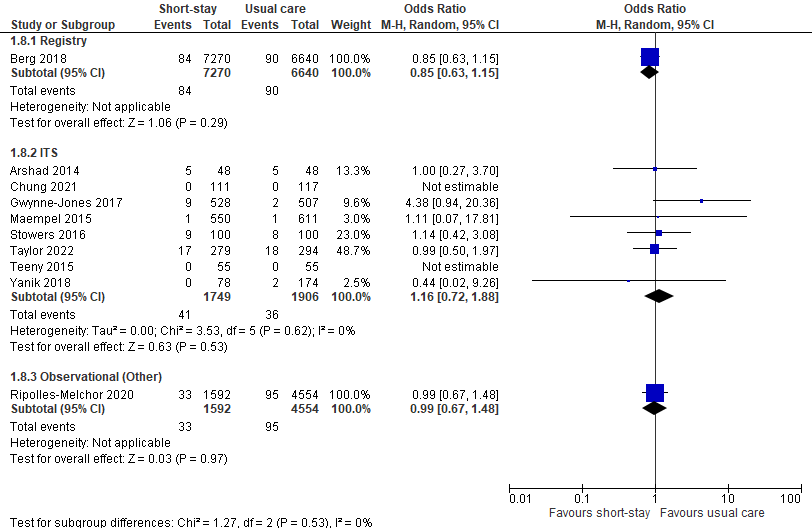
Wound Complications**

**
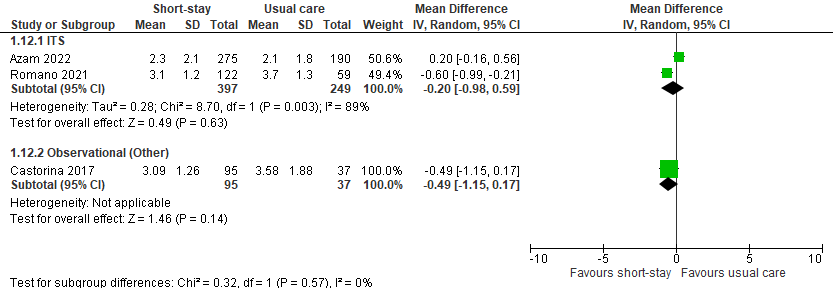
Blood Loss**

**
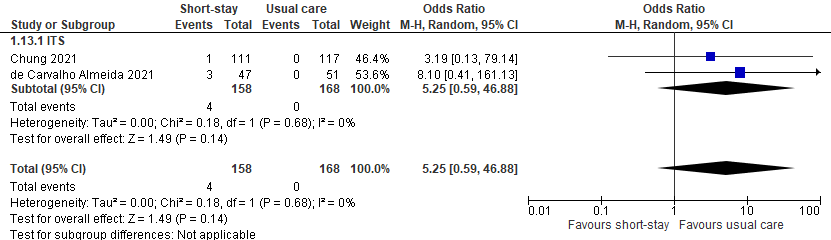
Periprosthetic Fracture**

**Emergency Department visits**


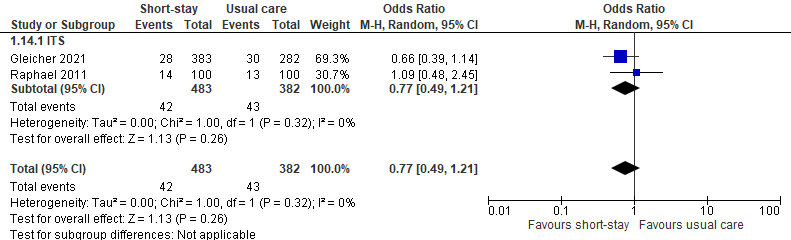


**
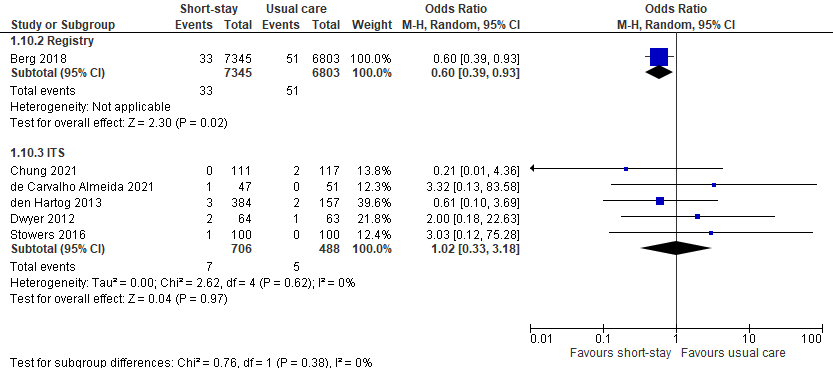
Dislocation**

**
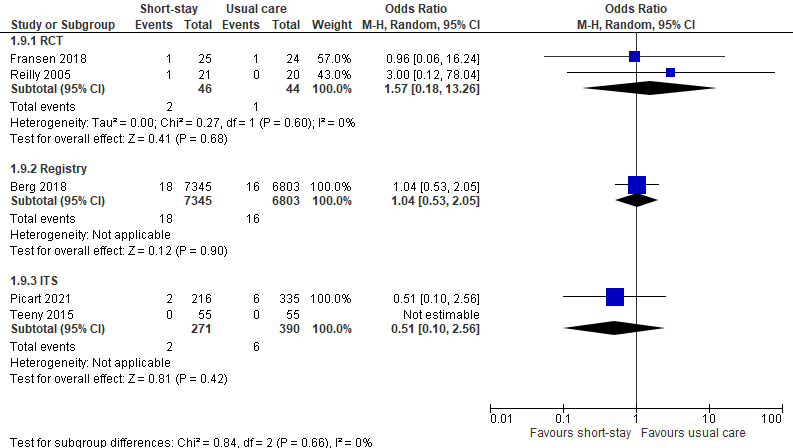
Stiffness and/or manipulation**
