# Supplementary material for: A systematic review and meta-analysis of short-stay programmes for total hip and knee replacement, focusing on safety and optimal patient selection
Source: BMC Med. 2023 Dec 21;21:511. doi: 10.1186/s12916-023-03219-5 (PMC10740291; doi:10.1186/s12916-023-03219-5)
Supplement: Supplementary file 5 — Additional file 5. [file 12916_2023_3219_MOESM5_ESM.docx]

**Supplementary File 5.** **Safety profile results reported in included studies but unable to be included in pooled analyses**

|  | Readmissions | Reoperations | Blood Loss | Complications | ED Visits | Mortality | Periprosthetic Fractures |
| --- | --- | --- | --- | --- | --- | --- | --- |
| Study | **Randomised Controlled Trials** | | | | | | |
| Petersen 2006 |  |  | Intraoperative Bleeding  Short-stay: 234.1 ml  Usual care: 387.9 ml  Postoperative Haemoglobin  Short-stay: mean 6.94  Usual care: mean 6.94 |  |  |  |  |
|  | **Registry Studies** | | | | | | |
| Berg 2018 | THR 30 Days  Short-stay: OR 1.2  95% CI 0.9, 1.5  THR 90 Days  Short-stay: OR 1.1  95% CI 0.9, 1.3  TKR 30 Days  Short-stay: OR 1.1  95% CI 0.9, 1.4  TKR 90 Days  Short-stay: OR 1.1  95% CI 0.9, 1.3 |  |  | THR 30 Days  Short-stay: OR 1.1  95% CI 0.9, 1.3  THR 90 Days  Short-stay: OR 1.1  95% CI 0.9, 1.2  TKR 30 Days  Short-stay: OR 1.1  95% CI 0.9, 1.3  TKR 90 Days  Short-stay: OR 1.2  95% CI 1.0, 1.4 |  |  |  |
| Berg 2021 |  |  |  |  |  | THR 90 Days  HR: 0.80  95% CI 0.55, 1.17  TKR 90 Days  HR: 0.69  95% CI 0.45, 1.07 |  |
|  | **Interrupted Time Series Studies** | | | | | | |
| Amlie et al 2016 |  | Reoperation Risk:  Aged >70  OR 1.710  95% CI 0.761, 3.838  p=0.194  Male  OR 2.899  95% CI 1.311, 6.410 p=0.009  Short-Stay  OR 3.315  95% CI 1.125, 9.772 p=0.030 |  |  |  |  |  |
| Arshad et al 2014 |  |  | Median Postop Hb  Short-stay: 11.5  95% CI 10.3, 12.3  Usual care: 10.6  95% CI 9.5, 11.4  p=<0.01  95% CI -1.80, -0.60 | Short-stay Yes: n=0  Short-stay No: n=48  No usual care figures were reported. |  |  |  |
| Azam et al 2022 |  |  | Mean (SD) Hb Drop  Short-stay: 2.3 (2.1)  Usual care: 2.1 (1.8) |  |  |  |  |
| Chung et al 2021 |  |  |  |  |  |  | THR  Short-stay: n=1 |
| de Carvalho Almeida 2021 |  |  |  |  |  |  | Short-stay: n=3, 6.4%  Usual care: n=0 |
| den Hartog et al 2013 | Short-stay: 4.4%  Usual care: 4.5%  p=0.7 | Short-stay: 2.9%  Usual care: 3.8%  p=0.5 |  | Short-stay: 7.8%  Usual care: 7.6% |  |  |  |
| *Featherall et al 2018 |  |  |  | Predictors  Gender  OR 0.951  95% CI 0.81, 1.12  p=0.534  Age  OR 1.005  95% CI 0.99, 1.01  p=0.209  BMI  OR 1.003  95% CI 0.99, 1.02  p=0.640  Diabetes  OR 0.124  95% CI 0.88, 1.43  p=0.340  Comorbidities  OR 1.158  95% CI 1.10, 1.22  p<0.001  White Race  OR 0.605  95% CI 0.48, 0.76  p<0.001  Public Insurance  OR 1.305  95% CI 1.07, 1.59  p=0.009  Self-Pay/Unknown  OR 1.061  95% CI 0.77, 1.45  p=0.714  High-Volume Hospital  OR 0.685  95% CI 0.54, 0.87  p=0.002  High-Volume Surgeon  OR 1.077  95% CI 0.89, 1.30  p=0.443 |  |  |  |
| Galbraith et al 2017 | 30 Days THR  p=0.098  90 Days THR  p=0.052  30 Days TKR  p=0.843  90 Days TKR  p=0.36 |  |  |  |  |  |  |
| Gleicher et al 2021 |  |  |  | Short-stay: 15.9%  Usual care: 95.3%  P<0.001 | Short-stay: 7.3%  Usual care: 12.9%  P=0.030 |  |  |
| Gwynne-Jones et al 2017 |  |  | THR  Short-stay: 17%  Usual care: 26%  p=0.18  TKR  Short-stay: 9%  Usual care: 9% |  |  |  |  |
| *Joo et al 2022 | Predictors  Change of Care  OR 0.88  95% CI 0.46, 1.65  SE 0.28  p=0.69  Age  OR 1.02  95% CI 0.99, 1.06  SE 0.02  p=0.20  Gender  OR 0.55  95% CI 0.29, 1.05  SE 0.18  p=0.07  Surgery Type  OR 0.97  95% CI 0.51, 1.86  SE 0.39  p=0.93  1^st^ Case of Day  OR 0.89  95% CI 0.48, 1.67  SE 0.29  p=0.72 |  |  | Predictors  Change of Care  OR 0.72  95% CI 0.43, 1.22  SE 0.19  p=0.22  Age  OR 1.03  95% CI 1.00, 1.07  SE 0.06  p=0.03  Gender  OR 0.64  95% CI 0.38, 1.07  SE 0.17  p=0.09  Surgery Type  OR 0.50  95% CI 0.30, 0.86  SE 0.14  p=0.01  1^st^ Case of Day  OR 1.45  95% CI 0.86, 2.45  SE0.39  p=0.16 |  |  |  |
| Larsen et al 2008 |  |  |  | THA  Short-stay: n=1 |  |  |  |
| Maempel et al 2015 |  |  | Change in Hb, Mean (SD)  Short-stay: -2.2g/dl (0.8)  Usual care: -1.8g/dl (0.7)  p=0.007 |  |  |  |  |
| Malviya et al 2011 |  |  | Short-stay: 9.8%  Usual care: 23%  p<0.001 |  |  |  |  |
| Picart et al 2021 |  |  | Transfusion Rate  Short-stay: 0%  Usual care: 5.87% |  |  |  |  |
| Raphael et al 2011 |  |  | Intraop Transfusion  Short-stay: 0  Usual care: 0  Postop Transfusion  Short-stay: 8%  Usual care: 8% |  | Short-stay: 14%  Usual care: 13% |  |  |
| Romano et al 2021 |  |  | Mean (SD) Hb Drop  Short-stay: 2.1 (1.2)  Usual care: 3.7 (1.3) |  |  |  |  |
| Savaridas et al 2013 |  |  |  |  |  | Survival Probability  KM 1 Month  Short-stay: 0.999  95% CI 0.025  Usual care: 0.995  95% CI 0.0013  KM 3 Months  Short-stay: 0.997  95% CI 0.0029  Usual care 0.992  95% CI 0.003 |  |
| Tasso et al 2022 |  |  | Hb at Discharge  Short-stay: 10.6 (1.4) g/dl  Usual care: 9.6 (1.2) g/dl  p=0.049  Hb at 30 Days  Short-stay: 13.7 (2.0) g/dl  Usual care: 12.5 (1.3) g/dl  Patients Req. Blood  Short-stay: 4%  Usual care: 8%  p=0.026 |  |  | Mortality  Short-stay: 0.1%  Usual care: 0.1% |  |
|  | **Other Observational Study Designs** | | | | | | |
| Reinhard et al 2023 |  |  |  | Nausea  Short-stay: 24.1%  Usual care: 25.1%  p=0.853  Dizziness  Short-stay: 32.5%  Usual care: 28.3%  p=0.298 |  |  |  |
| *Rippoles-Melchor et al 2019 |  |  | Postoperative Haemorrhage (n, %)  Short-stay: 31 (2.3%)  Usual care: 106 (5.1%)  OR 0.44  95% CI 0.29, -0.65 | Predictors  Age  OR 1.02  95% CI 1.01, 1.03  p<0.001  Frailty Score  OR 1.34  95% CI 1.25, 1.44  p<0.001  ASA III  OR 2.36  95% CI 1.62, 3.55  p<0.001  ASA IV  OR 4.50  95% CI 2.04, 9.47  p<0.001  Diabetes  OR 1.80  95% CI 1.22, 2.57  p=0.002  Heart Failure  OR 2.45  95% CI 1.65, 3.54  p<0.001  Atrial Fibrillation  OR 1.90  95% CI 1.46, 2.46  p<0.001  Kidney Disease  OR 1.81  95% CI 1.34, 2.41  p<0.001  Haemoglobin Level  OR 0.79  95% CI 0.75, 0.84  p<0.001  Blood Loss  OR 1.00  95% CI 1.00, 1.00  p<0.001  Tranexamic Acid  OR 0.56  95% CI 0.47, 0.66  p<0.001  Regional Anaesthesia  OR 0.74  95% CI 0.61, 0.89  p=0.001  Early Mobilization  OR 0.64  95% CI 0.53, 0.76  p<0.001  Insignificant Predictors: BMI, ASA I, ASA II, presurgical education and optimization, preoperative fasting, patient blood management, carb drink loading, avoiding long-acting sedatives, thromboprophylaxis, postop nausea and vomiting prophylaxis, active prevention of unintentional hypothermia, goal-directed fluid therapy, postop analgesia, postop glycaemic control, early feeding, epidural regional or local anaesthesia. |  |  |  |
| Wang et al 2023 |  |  | Mean (SD) Hb  Short-stay preop: 133.5 (16.1)  Day 3: 109.8 (15.5)  Usual care preop: 120.4 (20.0)  Day 3: 92.9 (16.9) |  |  |  |  |

CI: confidence interval; Hb: haemoglobin; KM: Kaplan Meier; OR: odds ratio; SD: standard deviation; SE: standard error; SS short-stay; TKR: total knee replacement; THR: total hip replacement.

* These studies looked at associations for the study sample overall and not specific to the short-stay and intervention groups
